# Subgroup Analysis in Pulmonary Hypertension-Specific Therapy Clinical Trials: A Systematic Review

**DOI:** 10.3390/jpm12060863

**Published:** 2022-05-25

**Authors:** Héctor Rodríguez-Ramallo, Nerea Báez-Gutiérrez, Remedios Otero-Candelera, Laila Abdel-kader Martín

**Affiliations:** 1 Hospital Pharmacy Department, Virgen del Rocio University Hospital, 41004 Seville, Spain; hector.rodriguez.sspa@juntadeandalucia.es (H.R.-R.); laila.abdelkader.sspa@juntadeandalucia.es (L.A.-k.M.); 2 Hospital Pharmacy Department, Reina Sofía University Hospital, 14004 Cordoba, Spain; 3 Pneumology Department, Virgen del Rocio University Hospital, 41004 Seville, Spain; remeotero@gmail.com

**Keywords:** pulmonary hypertension, subgroup analyses, randomized controlled trials, methodological limitations

## Abstract

Pulmonary hypertension (PH) treatment decisions are driven by the results of randomized controlled trials (RCTs). Subgroup analyses are often performed to assess whether the intervention effect will change due to the patient’s characteristics, thus allowing for individualized decisions. This review aimed to evaluate the appropriateness and interpretation of subgroup analyses performed in PH-specific therapy RCTs published between 2000 and 2020. Claims of subgroup effects were evaluated with prespecified criteria. Overall, 30 RCTs were included. Subgroup analyses presented: a high number of subgroup analyses reported, lack of prespecification, and lack of interaction tests. The trial protocol was not available for most RCTs; significant differences were found in those articles that published the protocol. Authors reported 13 claims of subgroup effect, with 12 claims meeting four or fewer of Sun’s criteria. Even when most RCTs were generally at low risk of bias and were published in high-impact journals, the credibility and general quality of subgroup analyses and subgroup claims were low due to methodological flaws. Clinicians should be skeptical of claims of subgroup effects and interpret subgroup analyses with caution, as due to their poor quality, these analyses may not serve as guidance for personalized care.

## 1. Introduction

Pulmonary hypertension (PH) is a rare disorder that may surge due to multiple clinical conditions or appear spontaneously without a clear cause [1]. Among other factors, the variety of etiologies of PH makes it an extremely complex disease; for this reason, a clinical classification was developed to group PH according to clinical presentation, findings, underlying conditions, and treatment [2]. PH is currently classified into five categories: group I pulmonary arterial hypertension, group II pulmonary hypertension due to left heart disease, group III pulmonary hypertension due to lung disease and/or hypoxemia, group IV chronic thromboembolic pulmonary hypertension, and group V pulmonary hypertension with unclear and/or multifactorial mechanisms.

As PH affects older patients disproportionally and may cause rapid deterioration and an increased risk of death, it is considered a major health issue, specifically in countries with older populations [3]. The current standards of PH treatment include drugs targeting endothelin-1, nitric oxide, and prostacyclin pathways. These treatments aim to promote vasodilation and avoid vascular remodeling [1]. However, the extending knowledge of the pathophysiology of the disease allows for the discovery of new targets [4,5]. An innovative approach is to restore the balance between the activation of the growth-promoting activin growth differentiation factor pathway and the growth-inhibiting bone morphogenetic protein receptors, with sotatercept showing promising results in a phase II randomized controlled trial (RCT) [6]. The choice of treatment for PH will vary according to the group of PH hypertension being treated, as therapies usually considered appropriate may even be harmful in a certain subgroup of patients [1].

PH treatment decisions are driven by the results of RCTs. Usually, only average results are reported in RCTs, and trial participants are often recruited from heterogeneous populations. However, clinicians ideally want more specific information to assist them in applying trial results to individual patients. Researchers conducting an RCT usually perform a subgroup analysis to assess whether the effect of the intervention will change due to the patient’s baseline characteristics, such as underlying pathologies, age, sex, or severity of the disease, which may allow for individualized decisions. Based on subgroup analysis results, researchers may report claims of subgroup effects. However, subgroup claims should be interpreted with caution, as misstatements about subgroup effects may result in patients being denied beneficial treatments, or even receiving treatments that may be ineffective or harmful [7,8,9].

The need for standards for the interpretation of subgroup analysis is crucial for treatment decisions in medical practice. Previous evaluations of RCT subgroup analyses have consistently documented poor-quality methods that burden its credibility. These reports showed suboptimal decisions in the design of the analyses, such as a lack of prespecification in trial protocols, adequate statistical methods, and biological rationale [10,11,12,13].

In order to provide tools that assist readers of medical literature in evaluating the credibility of subgroup analyses, explicit criteria were developed [14,15,16,17,18,19]. Recent tools to evaluate subgroup credibility were published, such as Schandelmeier S et al., 2020 [14] and Gil-Sierra MD et al., 2020 [15]. However, as far as we are concerned, the “10 criteria for assessing the credibility of a subgroup claim” [19] is the most reliable tool to assess confidence in a subgroup analysis, as the criteria were widely tested in several disciplines [10,11,12,13].

The central purpose of this study was to evaluate the appropriateness and interpretation of subgroup analyses performed in PH-specific therapy RCTs. In order to achieve our goals, the following aspects were studied:

- Description of subgroup analysis and claims of subgroup effects.
- Research characteristics of subgroup analysis.
- Analysis and interpretation of subgroup effects for primary outcomes.
- Assessment of the credibility of subgroup claims using the “10 criteria for assessing the credibility of a subgroup claim” [19].

## 2. Materials and Methods

### 2.1. Literature Search

This systematic review aims to summarize the available data to solve the following research questions, framed in the population, intervention, comparator, outcome, and study (PICOS) design framework: population: patients with PH; intervention: PH-specific therapy; comparison: studies with a comparator will be considered; outcomes: subgroup analysis; study design: randomized clinical trials.

The following groups of drugs were considered PH-specific therapy for this review:

- Phosphodiesterase type 5 inhibitors.
- Endothelin receptor antagonists.
- Prostacyclin analogues and prostacyclin receptor agonists.
- Calcium channel blockers.
- Guanylate cyclase stimulators.

A systematic search was conducted according to the preferred reporting items for a systematic review and meta-analysis (PRISMA) guidelines [20]. The systematic review protocol was registered with the prospective register for systematic review protocols (PROSPERO), registration number: CRD42021242265.

The search was conducted between January 2000 and December 2020 using vocabulary and keywords controlled by MeSH terms in the MEDLINE database to identify RCT-assessing PH-specific therapy for PH patients.

The search was performed in March 2021. The full literature search strategy is available in Supplemental File S1.

The following criteria were used for the trial selection:

Eligibility criteria:

- We considered all published PH-specific therapy RCTs on PH hypertension adults with subgroup analysis reported.

Exclusion criteria:

- Articles written in languages other than English, Spanish, and French.
- Post hoc analyses of previously published RCTs.
- Articles that were not available.
- Trials in which subgroup analysis credibility was impossible to evaluate due to missing data.

### 2.2. Study Screening and Selection

Two investigators independently checked the titles and abstracts of the search results using predefined inclusion criteria. The full text was accessed for all titles that seemed to meet the inclusion criteria or have uncertainties. Two reviewers, HRR and NBG, assessed whether the article met the selection criteria. Any disagreements were resolved through discussion or arbitration with the third reviewer, LAM.

### 2.3. Data Extraction

For data extraction, other sources included in the study were used (i.e., trial registration, published protocols, and online supplements). Data were extracted and entered into a structured Microsoft Excel (Redmond, WA, USA) database.

Eligible RCTs were evaluated to determine whether a subgroup analysis was reported. A *subgroup factor* was defined as a study variable by which the population may be categorized into different subgroups, i.e., sex, age, and the presence of a mutation. A *subgroup analysis* was defined as a specific analysis comparing two categories within a subgroup factor. For example, the analysis that compares the subgroups within the age factor >65 years vs. <65 years. A *subgroup effect* was defined as a difference in the magnitude of a treatment effect across a group of a study population [19]. For each RCT reporting subgroup analysis and subgroup claim, the following information was collected:

Trial characteristics: Information on the funding source, year and journal of publication, journal impact factor, clinical classification of PH [2], updated by the European Society of Cardiology and the European Respiratory Society Guidelines [1], center (multicentric or unicentric), trial design (parallel, crossover, or factorial), trial type (superiority, noninferiority, or equivalence), allocation concealment, blinding of patients, and the number of patients randomized. The primary endpoint was categorized according to whether the results were statistically significant and the type of outcome variable (time-to-event, binary, continuous, or count).

Reporting of subgroup analysis: Number of subgroup factors, type of subgroup factors (clinical factors or biomarkers), number of subgroup analyses and outcomes for subgroup analyses reported, forest plots used, whether it was a prespecified or post hoc subgroup, and the statistical method used to assess the heterogeneity of the treatment effect (descriptive only, subgroup *p* values and confidence interval or interaction test). When the trial protocol was available, the agreement on the number of subgroup factors, the number of subgroup analyses, and the prespecification of such analyses between the journal publication and the trial protocol were measured.

In order to assess possible differences in the quality of subgroup analyses reporting according to PH clinical groups, the variables were described separately for trials including patients in different clinical PH groups [2]. Claims of subgroup effects: The mode of presentation (abstract or text only) of subgroup claims, number of subgroup claims, subgroup variable (primary or secondary outcome), and the number of outcomes for subgroup claims were recorded. A subgroup effect was considered to be claimed when the authors stated in the abstract or discussion that the intervention effect differed between the categories of the subgroup variable. The claims of subgroup effects were classified according to the strength of the claim into three categories: strong claim, a claim of a likely effect, or suggestion of a possible effect based on Sun et al. classification (Supplemental File S2). To evaluate the credibility of subgroup claims for primary outcomes, “the 10 criteria for assessing the credibility of a subgroup claim” were applied pairwise (Supplemental File S3). If the subgroup claim met less than half the criteria, the credibility of this claim was considered low.

### 2.4. Assessment of Risk of Bias

The Cochrane Collaboration tool for assessing randomized trials [21] was used to evaluate the risk of bias in five dominions (randomization process, deviation from intended interventions, missing outcome data, measurement of the outcome, and selection of the reported result) and to present the results for each study across all dominions. Two independent reviewers evaluated the risk of bias. Possible disagreements between the reviewers were resolved by discussion or arbitration by a third reviewer when a consensus could not be reached.

### 2.5. Secondary Analyses

The quality of subgroup analysis reports during four time periods (2000–2004, 2005–2009, 2010–2014, and 2015–2019) were compared. This analysis aims to assess whether the methodology reported to perform subgroup analyses has improved over time.

### 2.6. Data Analysis

A descriptive analysis was developed. Continuous and categorical variables were presented as mean (range) and *n* (%), respectively.

For those RCTs that stated a subgroup effect without providing an interaction test, P interaction was calculated using the Joaquin Primo calculator [22] to verify that there was indeed statistical significance.

The inter-reviewer agreement for assessing the credibility of the subgroup claims was estimated by Cohen’s kappa coefficient.

## 3. Results

**Figure 1:**
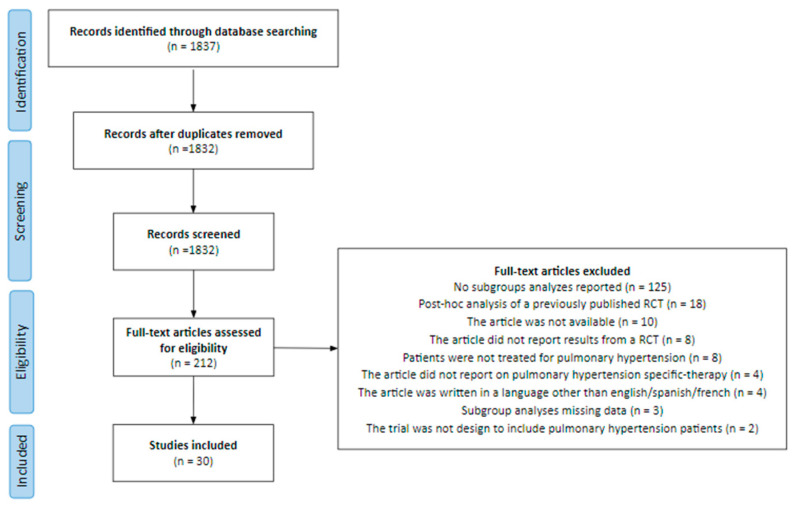
Flowchart of the screening of randomized clinical trials included in this analysis.

The initial literature search identified 1837 studies. After the first review by title or abstract and the deletion of duplicates, 185 articles were selected for full-text review. Finally, 30 papers were included (Figure 1). The excluded articles and the reasons for their exclusion are provided in the supplementary material (Supplemental File S4).

### 3.1. Trial Characteristics

The characteristics of the trials included in this study are listed in Table 1. The included publications reported data on 7765 randomized patients (median: 208; range: 52–1156).

Most studies were industry-funded (90%, *n* = 27). The most frequently selected journals for publication were The New England Journal of Medicine (*n* = 8) and Circulation (*n* = 4). Overall, 73% of the studies were published in high-impact journals (impact factor > 10).

The most common PH type explored was type 1 (*n* = 20). The stated primary endpoint was statistically significant in 63% (*n* = 19) of trials.

### 3.2. Subgroup Analyses

Characteristics of reported subgroup analyses are listed in Table 2. Subgroup analyses are mostly mentioned in the results and the discussion sections. Most trials, 57% (*n* = 17), did not clearly report the number of subgroup factors or subgroup analyses carried out. The remaining trials reported at least five subgroup factors or subgroup analyses in 37% (*n* = 11) and 40% (*n* = 12) of the trials, respectively. Subgroup analysis for more than one outcome was reported in 17% (*n* = 5) trials. Forest plots were used to report subgroup analysis data in 53% (*n* = 16) of the trials.

For 30% (*n* = 9) of trials, it was unclear whether subgroup analysis was pre-planned or post hoc; 47% (*n* = 14) of the trials were prespecified, 17% (*n* = 5) were post hoc, and 7% (*n* = 2) were prespecified and post hoc.

Only 37% (*n* = 11) of the trials used an interaction test to assess heterogeneity of the treatment effect; 33% (*n* =10) reported subgroup analysis without any statistical analysis.

The clinical trial protocol was available for 8 of the 30 RCTs included. Relevant differences were found for all 8 of the RCTs when comparing the trial protocol and the published manuscript:

- Subgroup analyses: Six RCTs reported fewer subgroup analyses than prespecified in the trial protocol. The remaining two RCTs reported subgroup analyses that were not prespecified in the trial protocol; in both cases, these analyses were characterized as prespecified in the published manuscript.
- Subgroup factors: The number of subgroup factors reported differed between the protocol and the published manuscript in seven cases: five RCTs reported fewer factors than those specified in the protocol. The remaining two added several subgroup factors that were not previously defined.
- Selective reports of subgroup analyses by outcome: There were differences in the number of subgroup analyses reported for the primary outcome in seven RCTs. In addition, in four trial protocols, the authors specified that subgroup analysis would be carried out for primary and secondary endpoints; however, the published manuscript only reported the subgroup analyses for the primary endpoint on three of these RCTs.

### 3.3. Claims of Subgroup Effects

Table 3 lists the characteristics of RCTs with subgroup claims. In 11 RCTs [23,24,25,26,27,28,29,30,31,32,33], the authors claim heterogeneity of treatment effect in at least one subject subgroup. Two RCTs each made two claims of subgroup differences [32,33]. Of the RCTs with claims of a subgroup effect, 4 out of 11 reached the primary endpoint, 5 did not, and for the rest, a clear primary endpoint was not defined. Only three RCTs provided interaction test results to prove a subgroup difference.

A total of 13 subgroup differences were claimed in 11 trials. The claims were classified as three (23%) strong claims, one (8%) claim of a likely effect, and nine (69%) suggestions of a possible effect.

Table 4 lists the 10 criteria to assess the credibility of subgroup claims as identified by strength.

### 3.4. Secondary Analyses

**Figure 2:**
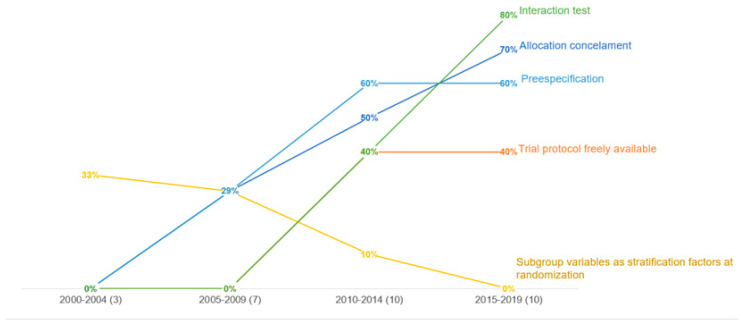
Evolution of subgroup analysis reporting.

Figure 2 shows the evolution of the quality of the subgroup analyses reported over four periods of time: 2000–2004, 2005–2009, 2010–2014, and 2015–2019.

An improvement was observed for most key methodological characteristics of PH-specific therapy RCTs over time, except for the use of subgroup variables as a stratification factor at randomization.

### 3.5. Risk of Bias

The risk of bias graphs within studies and across studies are available in the Supplemental Material (Supplemental File S5).

### 3.6. Inter-Reviewer Agreement across Reviewers

The inter-reviewer agreement for assessing the credibility of the subgroup claims was 0.88 (95% CI: 0.77–0.98), representing substantial to almost perfect agreement.

## 4. Discussion

Subgroup analyses have the potential to generate investigation hypotheses, identify baseline factors that may influence treatment efficacy or toxicity, and help clinicians make clinical decisions for personalized care. However, misusing subgroup analyses may also lead to spurious findings and misleading interpretations [34,35,36]. The most frequent methodological limitations of subgroup analyses in RCTs have been reported extensively: multiple testing of hypotheses, inadequate statistical power, inappropriate a priori specification, and a lack of biological rationale [7,8,37,38,39,40].

This systematic review found that the subgroup analyses in RCTs of PH-specific therapy are generally of low quality, despite being published primarily in high-impact factor journals. For most clinical trials, the study protocol was not available; therefore, it was challenging for reviewers to verify critical aspects such as the prespecification of the subgroup analyses. Furthermore, only one RCT had available the trial protocol among those claiming a subgroup effect. Of those studies for which the protocol was available, the subgroup analyses reported in the manuscript lacked description and were significantly different from those planned in the protocol.

Other factors that stand out among the methodological errors when performing subgroup analyses were identified as follows: a high number of subgroup analyses reported, a high number of post hoc analyses, and the lack of an interaction test to confirm the existence of subgroup effects. When multiple subgroup analyses are carried out, the results obtained should be interpreted with caution since the probability of obtaining a false positive can be significantly augmented [8]. This risk may be increased, especially if the hypothesis of the subgroup analysis was not prespecified [8,10,36]. The approximate calculated risk for a false-positive result for five subgroup analyses is 25%; however, it may increase as the number of subgroup analyses rises. We identified a median of six subgroup analyses reported among the RCTs evaluated in this review.

The prespecification of subgroup analysis is a frequent parameter measured in order to estimate methodological quality. For a subgroup analysis to be prespecified, it must be planned and documented before any examination of the data; this is based on the premise that a prespecified analysis usually follows a biological rationale. However, prespecification alone may not lead to solid subgroup analyses, as prespecified analyses may be based on unlikely and poorly formulated hypotheses [40]. In PH-specific therapy RCTs, 47% (14) of subgroup analyses were prespecified. In addition to the prespecification of the subgroup analyses, the correct direction of subgroup hypotheses must also be specified. For those claims in which the direction of the effect is not identified or is wrongly identified, their credibility could be reduced.

A common mistaken belief among authors is to claim a subgroup difference when a statistically significant effect is found in one subgroup but not in the other. One of the essential criteria to appropriately establish a claim of a subgroup effect is performing an interaction test [41]. The *p*-value of an interaction test provides information about the probability that the existence of a subgroup difference is due to an accidental finding or chance rather than an actual subgroup effect. In this review, we observed that only 38% of the RCTs performed an interaction test to confirm the existence of a subgroup effect. Of the 9 claims of subgroup difference identified in this study, 44% (*n* = 4) were based on a significant interaction test. We found mixed results when compared with similar studies in other clinical areas. Wallach et al. identified that among a sample of articles that made at least one claim in the abstract, 40% of the subgroups’ claims were based on an interaction test [42]. On the other hand, Khan et al. evaluated the quality of subgroup analyses in heart failure RCTs, reporting 70% of claims were based on significant interaction tests [43].

Most of the studies included in this review were industry-funded (90%), which potentially influenced our results. The funding source of clinical trials may play a role in the quality of the reports of subgroup analyses; industry-funded RCTs are more likely to report subgroup analyses [44,45,46], even when an overall treatment effect for a primary outcome could not be proven [44]. Industry funding was also correlated with suboptimal reporting of subgroup effects; often, the subgroup hypotheses were not prespecified, and the use of an interaction test was rare [44,46]. This is consistent with our findings in this primarily industry-funded sample of RCTs as, among the articles that claimed difference of subgroup effect, only four reached the primary endpoint.

Previous studies have found that the methodological quality reported in the methods sections of published articles is lacking compared to study protocols [45,47,48], finding high-quality studies to be poorly reported. Protocols provide a complete insight into the analysis methods utilized in RCTs. It is recommended to publish trial protocols all together with the publication of the RCT and its publication in clinical trial registries, thus providing the reader with a transparent and complete description of the prespecified methods. However, several studies have found that RCT protocols are often not freely available [45,49]; this is consistent with our findings. Only 7 out of 30 RCTs provided the study protocol, and discrete growth in protocol publishing was observed during the studied period.

The fact that protocols are not systematically accessible is alarming; even when voluntarily published, discrepancies within journal publications are relatively frequent when reporting study outcomes [50,51,52,53,54,55,56,57,58]. Similarly, a high number of inconsistencies between protocols and publications were described in several methodological characteristics of subgroup analyses, including omitted prespecified analyses, interaction tests, prespecification of subgroup analyses, and minor differences for the anticipated direction of the effect [45,58]. Due to these prevalent discrepancies, the credibility of subgroup methods may be questionable if the study protocol is not accessible. Our findings coincide with previous reports; few studies published the protocol in either the journal publication or clinical trial registries. Among 14 studies that reported a prespecified subgroup analysis, only half published the study’s protocol. Furthermore, a third of the studies did not report clearly whether the subgroup analysis was prespecified or post hoc; in none of these cases was the protocol freely available.

Despite the methodological limitations of subgroup analyses in RCTs being increasingly recognized, a review of 437 randomly selected RCTs published in high-impact journals found a decrease in the appropriateness of reporting subgroup analyses from 2007 to 2014 [46]. In contrast with these results, we observed an improvement in most methodological characteristics of PH-specific therapy RCTs; a priori specification, forest plot utilization, and interaction tests improved from 2002 to 2019. However, a decline in subgroup variables set as stratification factors during randomization was observed. When a particular characteristic is known to influence the trial outcome, it should be used as a stratification factor at randomization; thus, the decrease adds to the hypothesis that most subgroup analyses, even when prespecified, are exploratory. 

Claims of a subgroup effect are frequent in RCT reports. Several systematic reviews and analyses have shown that authors report a difference in treatment effects between patient subgroups in 40–60% of all RCTs reporting subgroup analyses [10,40,59]. Few systematic reviews have described a relatively low number of subgroup claims [11,43]. Our results, however, are in line with the latest reviews. We found that PH-specific therapy RCTs reported claims of subgroup effect on 27% (*n* = 9) of RCTs reporting subgroup analyses. Fewer subgroup claims may indicate that authors were cautious in their reporting of RCT analyses.

### 4.1. Strengths

To our knowledge, this is the first systematic review of the credibility of subgroup analyses and subgroup effect claims reported on PH-specific therapy RCTs. A rigorous systematic method was employed. Standardized criteria were used in order to assess the credibility of subgroup claims.

### 4.2. Limitations

This study has some limitations. First, although we used a scale to determine the credibility of the claims, the sun criteria were not designed to provide a score; therefore, the later interpretation of its results was not without subjectivity.

Secondly, when assessing the strength of a claim, there is an undeniable subjective value in interpreting the claims of the authors. However, the pairwise work and the high agreement in the results of both researchers suggest that this limitation was not significant.

Third, in most studies, we could not find the study protocols. In many cases, we could not know whether the published results corresponded to the initially defined objectives; this limited our ability to judge the credibility of subgroup claims. For this purpose, authors must provide detailed information about the conduct and results of a subgroup analysis.

### 4.3. Implications for Policy to Improve the Reporting of Subgroup Analyses

Although the methodological limitations of subgroup analyses are consistently reported in the literature, similar mistakes are carried out when conducting and reporting subgroup analyses in recent RCTs. As improvement measures to change the current state of subgroup analyses, we propose the following:

Firstly, subgroup analyses should be prespecified and documented in trial registries. Secondly, scientific journals should request authors to make the study protocol accessible to reviewers and readers as a requirement for publishing the results of RCTs. Thirdly the use of guidelines or tools for the correct publication of subgroup analyses should be enforced. Fourthly, researchers should be cautious when claiming subgroup differences, even when a robust methodology for subgroup analyses was followed.

## 5. Conclusions

Due to methodological flaws, subgroup analyses in PH-specific therapies are of poor quality. Overall, the credibility of subgroup claims was considered low, with most claims not meeting critical criteria. Therefore, clinicians should be skeptical of claims of subgroup effects and interpret subgroup analyses with caution, as due to their poor quality, these analyses may not serve as guidance for personalized care.

## Figures and Tables

**Table 1:** Characteristics of trials included.

Variable		Nº Trials (*n* = 30)	%
Funding source	Industry	27	90
	Non-industry	2	7
	Non specify	1	3
Year of publication	2000–2004	3	10
	2005–2009	7	23
	2010–2014	10	33
	2015–2019	10	33
Journal	Chest	2	7
	Circulation	4	13
	European Heart Journal	2	7
	Journal of the American College of Cardiology	2	7
	The Lancet Respiratory Medicine	2	7
	The New England Journal of Medicine	8	27
	Others	10	33
Journal impact factor	<10	8	27
	>10	22	73
Clinical PH classification	Group 1	20	67
	Group 2	3	10
	Group 3	2	7
	Group 4	3	10
	Any	2	7
Centre	Multicentric	27	90
	Unicentric	2	7
	Not specified	1	3
Trial design	Parallel	30	100
	Superiority	30	100
Allocation concealment	Yes	14	47
	No	1	3
	Unclear	15	50
Blinding	Open label	1	3
	Double-blinded	28	93
	Not specified	1	3
Protocol freely available	Yes	8	27
	No	22	67
Nº patients randomized ^1^	Total	7765	
	Median (range)	208 (52–1156)	
Nº arms	Median (range)	2 (2–5)	
Type of primary endpoint ^1^	Time-to-event	5	17
	Binary	2	7
	Continuous	23	77
Trial met the primary endpoint ^1^	Yes	19	63
	No	8	27

PH: Pulmonary Hypertension. ^1^ Extension trials were excluded from the descriptive analysis.

**Table 2:** Characteristics of subgroup analysis reporting.

Reporting of Subgroup Analysis		Group 1 (*n* = 20) ^1^	Group 2 (*n* = 3) ^1^	Group 3 (*n* = 2) ^1^	Group 4 (*n* = 3) ^1^	Any (*n* = 2) ^1^	All Trials (*n* = 30)
Mode of presentation	Abstract	2	-	-	-	1	3
	Methods	8	1	1	1	-	11
	Results	17	3	2	3	2	27
	Discussion	12	3	2	1	1	19
	Supplementary Material	6	1	-	1	-	8
Nº subgroup factors	2–4	1	-	-	-	1	2
	5–10	7	1	1	1	-	10
	>10	1	-	-	-	-	1
	Unclear	11	2	1	2	1	17
	Median (range)						6 (2–17)
Nº subgroup analysis reported	2–4	-	-	-	-	1	1
	5–10	8	1	1	1	-	11
	>10	1	-	-	-	-	1
	Unclear	11	2	1	2	1	17
	Median (range)						7 (2–36)
Nº subgroup outcomes	1	15	3	2	3	-	23
	2–5	2	-	-	-	1	3
	>5	2	-	-	-	-	2
	Unclear	2	-	-	-	-	2
	Median (range)						1 (1–12)
Forest plot	Yes	9	2	2	2	1	16
	No	11	1	-	1	1	14
Prespecified or post hoc	Prespecified	7	1	2	3	1	14
	Post hoc	5	-	-	-	-	5
	Prespecified and post hoc	2	-	-	-	-	2
	Unclear	6	2	-	-	1	9
Statistical method	Descriptive	5	2	-	2	1	10
	Subgroups P or CI	5	-	1	-	-	6
	Interaction test	8	1	1	1	-	11
	Unclear	2	-	-	-	1	3
Subgroup claim	Yes	9	1	-	-	1	11
	No	11	2	2	3	1	19

^1^ Patients included in the randomized controlled trials were classified according to the five pulmonary hypertension clinical groups [2]. CI: confidence interval.

**Table 3:** Characteristics of trials with claims of subgroup differences.

Claims of Subgroup Difference		Trials (*n* = 11)	%
Mode of presentation	Abstract	4	36
	Text only	7	64
Nº subgroup claims	1	9	82
	2	2	18
Subgroup variable	Primary endpoint	11	100
Forest plot	Yes	2	18
	No	9	82
Nº subgroup analysis	1–4	0	0
	5–10	2	18
	>10	1	9
	Unclear	8	73
	Median (range)	7 (7–12)	
Nº of outcomes for subgroup claims	1	8	73
	2–5	1	9
	>5	1	9
	Unclear	1	9
	Median (range)	1 (1–12)	
Statistical methods for subgroup analyses	Descriptive	3	27
	Subgroups P or CI	5	46
	Interaction test	3	27
Prespecified/post hoc	Prespecified	3	27
	Post hoc	4	36
	Prespecified and post hoc	1	9
	Unclear	3	27
Protocol was freely available	Yes	1	9
	No	10	91

**Table 4:** Claims meeting Sun’s et al. subgroup criteria for primary outcomes.

Criteria	Strong Claim (*n* = 3)	Claim of Likely Effect (*n* = 1)	Suggestion of Effect (*n* = 9)	All Claims (*n* = 13)
Subgroup variable as a baseline characteristic ^1^	3 (100%)	1 (100%)	9 (100%)	13 (100%)
Subgroup variable a stratification factor at randomization	0 (0%)	1 (100%)	2 (22%)	3 (23%)
Subgroup hypothesis specified a priori	0 (0%)	0 (0%)	3 (33%)	3 (23%)
A small number of hypothesized effects tested (</=5)	0 (0%)	0 (0%)	0 (0%)	0 (0%)
Significant interaction test (*p* < 0.05) ^2^	0 (0%)	0 (0%)	4 (45%)	4 (31%)
Independence of interaction ^1^	-	-	-	-
Direction of the subgroup effect correctly prespecified?	1 (33%)	0 (0%)	0 (0%)	1 (8%)
Subgroup effect consistency across studies	2 (66%)	0 (0%)	6 (67%)	8 (62%)
Subgroup effect consistent across related outcomes	-	-	-	-
Compelling indirect evidence	1 (33%)	0 (0%)	5 (56%)	6 (46%)

^1^ Two trials claimed two subgroup claims each. ^2^ For those RCT that stated a subgroup effect without providing an interaction test, P interaction was calculated using the Joaquin Primo calculator [22] to verify that there was indeed statistical significance.

## Data Availability

The dataset is available on request from the corresponding author.

## Supplementary Materials

The following are available online at https://www.mdpi.com/article/10.3390/jpm12060863/s1, Supplemental File S1: database search strategy; Supplemental File S2: criteria to assess the strength of subgroup claims; Supplemental File S3: criteria to assess the credibility of subgroup claims; Supplemental File S4: excluded articles; Supplemental File S5: risk of bias of the included articles.

## References

[1] Galiè N., Humbert M., Vachiery J.-L., Gibbs S., Lang I., Torbicki A., Simonneau G., Peacock A., Noordegraaf A.V., Beghetti M. (2016). 2015 ESC/ERS Guidelines for the diagnosis and treatment of pulmonary hypertension: The Joint Task Force for the Diagnosis and Treatment of Pulmonary Hypertension of the European Society of Cardiology (ESC) and the European Respiratory Society (ERS): Endorsed by: Association for European Paediatric and Congenital Cardiology (AEPC), International Society for Heart and Lung Transplantation (ISHLT). Eur Heart J..

[2] Simonneau G., Gatzoulis M.A., Adatia I., Celermajer D., Denton C., Ghofrani A., Sanchez M.A.G., Kumar R.K., Landzberg M., Machado R.F. (2013). Updated Clinical Classification of Pulmonary Hypertension. J. Am. Coll. Cardiol..

[3] Hoeper M.M., Humbert M., Souza R., Idrees M., Kawut S.M., Sliwa-Hahnle K., Jing Z.-C., Gibbs J.S.R. (2016). A global view of pulmonary hypertension. Lancet Respir. Med..

[4] Bisserier M., Pradhan N., Hadri L. (2020). Current and emerging therapeutic approaches to pulmonary hypertension. Rev. Cardiovasc. Med..

[5] Mercurio V., Bianco A., Campi G., Cuomo A., Diab N., Mancini A., Parrella P., Petretta M., Hassoun P.M., Bonaduce D. (2019). New Drugs, Therapeutic Strategies, and Future Direction for the Treatment of Pulmonary Arterial Hypertension. Curr. Med. Chem..

[6] Humbert M., McLaughlin V., Gibbs J.S.R., Gomberg-Maitland M., Hoeper M.M., Preston I.R., Souza R., Waxman A., Subias P.E., Feldman J. (2021). Sotatercept for the Treatment of Pulmonary Arterial Hypertension. N. Engl. J. Med..

[7] Wittes J. (2009). On Looking at Subgroups. Circulation.

[8] Wang R., Lagakos S.W., Ware J.H., Hunter D.J., Drazen J.M. (2007). Statistics in Medicine—Reporting of Subgroup Analyses in Clinical Trials. N. Engl. J. Med..

[9] Koch A., Framke T. (2014). Reliably Basing Conclusions on Subgroups of Randomized Clinical Trials. J. Biopharm. Stat..

[10] Sun X., Briel M., Busse J.W., You J.J., Akl E.A., Mejza F., Bala M.M., Bassler D., Mertz D., Diaz-Granados N. (2012). Credibility of claims of subgroup effects in randomized controlled trials: Systematic review. BMJ.

[11] Báez-Gutiérrez N., Rodríguez-Ramallo H., Flores-Moreno S., Martín L.A. (2020). Subgroup analysis in haematologic malignancies phase III clinical trials: A systematic review. Br. J. Clin. Pharmacol..

[12] Saragiotto B.T., Maher C.G., Moseley A.M., Yamato T., Koes B.W., Sun X., Hancock M. (2016). A systematic review reveals that the credibility of subgroup claims in low back pain trials was low. J. Clin. Epidemiol..

[13] Paquette M., Alotaibi A.M., Nieuwlaat R., Santesso N., Mbuagbaw L. (2019). A meta-epidemiological study of subgroup analyses in cochrane systematic reviews of atrial fibrillation. Syst. Rev..

[14] Schandelmaier S., Briel M., Varadhan R., Schmid C.H., Devasenapathy N., Hayward R.A., Gagnier J., Borenstein M., van der Heijden G.J., Dahabreh I.J. (2020). Development of the Instrument to assess the Credibility of Effect Modification Analyses (ICEMAN) in randomized controlled trials and meta-analyses. Can. Med. Assoc. J..

[15] Gil-Sierra M.D., Fenix-Caballero S., Kader-Martin L.A., Fraga-Fuentes M.D., Sánchez-Hidalgo M., De La Lastra-Romero C.A., Alegre-del Rey E.J. (2019). Checklist for clinical applicability of subgroup analysis. J. Clin. Pharm. Ther..

[16] Oxman A.D., Guyatt G.H. (1992). A consumer’s guide to subgroup analyses. Ann. Intern. Med..

[17] Sun X., Briel M., Walter S.D., Guyatt G.H. (2010). Is a subgroup effect believable? Updating criteria to evaluate the credibility of subgroup analyses. BMJ.

[18] Sun X., Ioannidis J.P., Agoritsas T., Alba A.C., Guyatt G. (2014). How to use a subgroup analysis: Users’ guide to the medical literature. JAMA.

[19] Sun X., Briel M., Busse J.W., Akl E.A., You J.J., Mejza F., Bala M., Diaz-Granados N., Bassler D., Mertz D. (2009). Subgroup Analysis of Trials Is Rarely Easy (SATIRE): A study protocol for a systematic review to characterize the analysis, reporting, and claim of subgroup effects in randomized trials. Trials.

[20] Moher D., Liberati A., Tetzlaff J., Altman D.G., Prisma Group (2010). Preferred reporting items for systematic reviews and meta-analyses: The PRISMA statement. Int. J. Surg..

[21] Higgins J.P., Thomas J., Chandler J., Cumpston M., Li T., Page M.J., Welch V.A. (2019). Cochrane Handbook for Systematic Reviews of Interventions Version 6.0 (Updated July 2019).

[22] Primo J., Escrig J. (2008). MetaSurv: Excel Calculator for Survival Meta-Analyzes. http://www.redcaspe.org/herramientas/descargas/MetaSurv.xls.

[23] Galiè N., Humbert M., Vachiéry J.-L., Vizza C., Kneussl M., Manes A., Sitbon O., Torbicki A., Delcroix M., Naeije R. (2002). Effects of beraprost sodium, an oral prostacyclin analogue, in patients with pulmonary arterial hypertension: A randomized, double-blind, placebo-controlled trial. J. Am. Coll. Cardiol..

[24] Olschewski H., Simonneau G., Galie N., Higenbottam T., Naeije R., Rubin L.J., Nikkho S., Speich R., Hoeper M., Behr J. (2002). Inhaled Iloprost for Severe Pulmonary Hypertension. N. Engl. J. Med..

[25] Simonneau G., Rubin L.J., Galie N., Barst R.J., Fleming T.R., Frost A.E., Engel P.J., Kramer M.R., Burgesset G., Collings L. (2008). Addition of sildenafil to long-term intravenous epoprostenol therapy in patients with pulmonary arterial hypertension: A randomized trial. Ann. Intern. Med..

[26] Benza R.L., Barst R.J., Galie N., Frost A., Girgis R.E., Highland K.B., Strange C., Black C.M., Badesch D.B., Rubin L. (2008). Sitaxsentan for the treatment of pulmonary arterial hypertension: A 1-year, prospective, open-label observation of outcome and survival. Chest.

[27] Barst R.J., Oudiz R.J., Beardsworth A., Brundage B.H., Simonneau G., Ghofrani A., Sundin D.P., Galie N. (2011). Tadalafil monotherapy and as add-on to background bosentan in patients with pulmonary arterial hypertension. J. Heart Lung Transplant..

[28] Ghofrani H.-A., Galie N., Grimminger F., Grünig E., Humbert M., Jing Z.-C., Keogh A.M., Langleben D., Kilama M.O., Fritsch A. (2013). Riociguat for the Treatment of Pulmonary Arterial Hypertension. N. Engl. J. Med..

[29] Tapson V.F., Jing Z.C., Xu K.F., Pan L., Feldman J., Kiely D.G., Kotlyar E., McSwain C.S., Laliberte K., Arneson C. (2013). Oral treprostinil for the treatment of pulmonary arterial hypertension in patients receiving background endothelin receptor antagonist and phosphodiesterase type 5 inhibitor therapy (the FREEDOM-C2 study): A randomized controlled trial. Chest.

[30] Hoendermis E.S., Liu L.C., Hummel Y.M., van der Meer P., de Boer R.A., Berger R.M.F., van Veldhuisen D.J., Voors A.A. (2015). Effects of sildenafil on invasive haemodynamics and exercise capacity in heart failure patients with preserved ejection fraction and pulmonary hypertension: A randomized controlled trial. Eur Heart J..

[31] Chang H.-J., Song S., Chang S.-A., Kim H.-K., Jung H.-O., Choi J.-H., Lee J.S., Kim K.-H., Jeong J.-O., Lee J.H. (2019). Efficacy and Safety of Udenafil for the Treatment of Pulmonary Arterial Hypertension: A Placebo-controlled, Double-blind, Phase IIb Clinical Trial. Clin. Ther..

[32] McLaughlin V., Channick R.N., Ghofrani H.-A., Lemarié J.-C., Naeije R., Packer M., Souza R., Tapson V.F., Tolson J., Al Hiti H. (2015). Bosentan added to sildenafil therapy in patients with pulmonary arterial hypertension. Eur. Respir. J..

[33] Vizza C.D., Jansa P., Teal S., Dombi T., Zhou D. (2017). Sildenafil dosed concomitantly with bosentan for adult pulmonary arterial hypertension in a randomized controlled trial. BMC Cardiovasc. Disord..

[34] Izem R., Liao J., Hu M., Wei Y., Akhtar S., Wernecke M., MaCurdy T.E., Kelman J., Graham D.J. (2020). Comparison of propensity score methods for pre-specified subgroup analysis with survival data. J. Biopharm. Stat..

[35] Kent D.M., Paulus J.K., Van Klaveren D., D’Agostino R., Goodman S., Hayward R., Ioannidis J.P., Patrick-Lake B., Morton S., Pencina M. (2019). The Predictive Approaches to Treatment effect Heterogeneity (PATH) Statement. Ann. Intern. Med..

[36] Assmann S.F., Pocock S.J., Enos L.E., Kasten L.E. (2000). Subgroup analysis and other (mis) uses of baseline data in clinical trials. Lancet.

[37] Brookes S.T., Whitely E., Egger M., Smith G.D., Mulheran P.A., Peters T.J. (2004). Subgroup analyses in randomized trials: Risks of subgroup-specific analyses; power and sample size for the interaction test. J. Clin. Epidemiol..

[38] Feinstein A.R. (1998). The Problem of Cogent Subgroups: A Clinicostatistical Tragedy. J. Clin. Epidemiol..

[39] Alegre del Rey E.J., Gil Sierra M.D., Alarcón de la Lastra Romero C., Sánchez Hidalgo M. (2021). Remdesivir and mortality reduction in COVID-19 patients: A systematized subgroup analysis of clinical trials. Farm. Hosp..

[40] Pharoah P. (2012). Response to Credibility of claims of subgroup effects in randomized controlled trials: Systematic review. BMJ.

[41] Sainani K. (2010). Misleading Comparisons: The Fallacy of Comparing Statistical Significance. PM&R.

[42] Wallach J.D., Sullivan P.G., Trepanowski J.F., Sainani K.L., Steyerberg E.W., Ioannidis J.P.A. (2017). Evaluation of Evidence of Statistical Support and Corroboration of Subgroup Claims in Randomized Clinical Trials. JAMA Intern. Med..

[43] Khan M.S., Irfan S., Siddiqi T.J., Greene S.J., Anker S.D., Sreenivasan J., Friede T., Tahhan A.S., Vaduganathan M., Fonarow G.C. (2020). Reporting and interpretation of subgroup analyses in heart failure randomized controlled trials. ESC Heart Fail..

[44] Sun X., Briel M., Busse J.W., You J.J., Akl E.A., Mejza F., Bala M.M., Bassler D., Mertz D., Diaz-Granados N. (2011). The influence of study characteristics on reporting of subgroup analyses in randomized controlled trials: Systematic review. BMJ.

[45] Kasenda B., Schandelmaier S., Sun X., Von Elm E., You J., Blümle A., Tomonaga Y., Saccilotto R., Amstutz A., Bengough T. (2014). Subgroup analyses in randomized controlled trials: Cohort study on trial protocols and journal publications. BMJ.

[46] Gabler N.B., Duan N., Raneses E., Suttner L., Ciarametaro M., Cooney E., Dubois R.W., Halpern S.D., Kravitz R.L. (2016). No improvement in the reporting of clinical trial subgroup effects in high-impact general medical journals. Trials.

[47] Mhaskar R., Djulbegovic B., Magazin A., Soares H.P., Kumar A. (2012). Published methodological quality of randomized controlled trials does not reflect the actual quality assessed in protocols. J. Clin. Epidemiol..

[48] Soares H.P., Daniels S., Kumar A., Clarke M., Scott C., Swann S., Djulbegovic B. (2004). Bad reporting does not mean bad methods for randomized trials: Observational study of randomized controlled trials performed by the radiation therapy oncology group. BMJ.

[49] Chan A.W., Hróbjartsson A., Jørgensen K.J., Gøtzsche P.C., Altman D.G. (2008). Discrepancies in sample size calculations and data analyses reported in randomized trials: Comparison of publications with protocols. BMJ.

[50] Chan A.W., Hróbjartsson A., Haahr M.T., Gøtzsche P.C., Altman D.G. (2004). Empirical evidence for selective reporting of outcomes in randomized trials: Comparison of protocols to published articles. JAMA.

[51] Hahn S., Williamson P.R., Hutton J.L. (2002). Investigation of within-study selective reporting in clinical research: Follow-up of applications submitted to a local research ethics committee. J. Eval. Clin. Pract..

[52] Chan A.W., Krleža-Jerić K., Schmid I., Altman D.G. (2004). Outcome reporting bias in randomized trials funded by the Canadian Institutes of Health Research. Cmaj.

[53] Turner E.H., Matthews A.M., Linardatos E., Tell R.A., Rosenthal R. (2008). Selective publication of antidepressant trials and its influence on apparent efficacy. N. Engl. J. Med..

[54] von Elm E.B., Röllin A., Blümle A., Huwiler K., Witschi M., Egger M. (2008). Publication and non-publication of clinical trials: Longitudinal study of applications submitted to a research ethics committee. Swiss Med. Wkly..

[55] Mathieu S., Boutron I., Moher D., Altman D.G., Ravaud P. (2009). Comparison of registered and published primary outcomes in randomized controlled trials. JAMA.

[56] Al-Marzouki S., Roberts I., Evans S., Marshall T. (2008). Selective reporting in clinical trials: Analysis of trial protocols accepted by The Lancet. Lancet.

[57] Ross J.S., Mulvey G.K., Hines E.M., Nissen S.E., Krumholz H.M. (2009). Trial publication after registration in ClinicalTrials.Gov: A cross-sectional analysis. PLoS Med..

[58] Li G., Abbade L.P., Nwosu I., Jin Y., Leenus A., Maaz M., Wang M., Bhatt M., Zielinski L., Sanger N. (2018). A systematic review of comparisons between protocols or registrations and full reports in primary biomedical research. BMC Med. Res. Methodol..

[59] Vidic A., Chibnall J.T., Goparaju N., Hauptman P.J. (2016). Subgroup analyses of randomized clinical trials in heart failure: Facts and numbers. ESC Heart Fail..

