# Supplementary material for: Subgroup Analysis in Pulmonary Hypertension-Specific Therapy Clinical Trials: A Systematic Review
Source: J Pers Med. 2022 May 25;12(6):863. doi: 10.3390/jpm12060863 (PMC9224970; doi:10.3390/jpm12060863)
Supplement: Supplementary file 1 [file jpm-12-00863-s001.zip › Supplemental File S1.pdf]

Supplemental File S1. Full search strategy

Full search strategy
1- exp Hypertension, Pulmonary/
2- PAH.tw.
3- (pulmonary adj3 hypertension).tw.
4- exp Randomized Controlled Trial/
5- (randomized controlled trial$ or randomised controlled trial$).mp. [mp = title, original title, abstract, name of substance word, subject heading word]
6- (randomized trial$ or randomised trial$).mp. [mp = title, original title, abstract, name of substance word, subject heading word]
7- (randomized clinical trial$ or randomised clinical trial$).mp. [mp = title, original title, abstract, name of substance word, subject heading word]
8- 4 or 5 or 6 or 7
9- 1 or 2 or 3
10- 8 and 9
11- limit 10 to (humans)
