# Supplementary material for: Subgroup Analysis in Pulmonary Hypertension-Specific Therapy Clinical Trials: A Systematic Review
Source: J Pers Med. 2022 May 25;12(6):863. doi: 10.3390/jpm12060863 (PMC9224970; doi:10.3390/jpm12060863)
Supplement: Supplementary file 1 [file jpm-12-00863-s001.zip › Supplemental File S2.pdf]

Supplemental File S2. Criteria for judging the strength of a subgroup claim

Criteria for judging the strength of a subgroup claim[1].			
	**Reasonably strong claim of a definitive effect**	**Claim of a likely effect**	**Suggestion of a possible effect**
1. Did the investigators claim the effect in the abstract?	Yes	Possible	No
2. Did the investigators claim the effect in the conclusion of abstract?	Possible*	No	No
3. Did the investigators claim the effect in the discussion?	Yes	Possible	Yes
4. Did the investigators use the descriptive words (e.g., appear/seem to be, may, and might) to soften their statements of the claims?	No	Possible	Possible
5. Did the investigators use descriptive words (e.g., particular and special) to strengthen the statement of the claims?	Possible	No	No
6. Were the authors obviously cautious about the apparent subgroup effect? (e.g., they stated the subgroup effect did not meet some of important criteria to believe a subgroup effect)	No	Some caution possible	Yes
7. Did the investigators indicate the apparent effects need to be explored in the future studies (i.e., hypothesis generating)?	No	Possible say desirable to confirm	No

Reference:

Sun X, Briel M, Busse JW, et al. Subgroup Analysis of Trials Is Rarely Easy (SATIRE): a study protocol for a systematic review to characterize the analysis, reporting, and claim of subgroup effects in randomized trials. Trials. 2009;10:101. Published 2009 Nov 9. doi:10.1186/1745-6215-10-101
