# Supplementary material for: Subgroup Analysis in Pulmonary Hypertension-Specific Therapy Clinical Trials: A Systematic Review
Source: J Pers Med. 2022 May 25;12(6):863. doi: 10.3390/jpm12060863 (PMC9224970; doi:10.3390/jpm12060863)
Supplement: Supplementary file 1 [file jpm-12-00863-s001.zip › Supplemental File S3.pdf]

Supplemental File S3. Criteria to assess the credibility of subgroup claims

Criteria to assess the credibility of subgroup claims[2]		
**Criterion**	**Description of criteria**	**Coding**
**Design**		
1. Is the subgroup variable a characteristic measured at baseline?	Subgroup variables measured after randomisation might be influenced by the tested interventions. The apparent difference of treatment effect between subgroups can be explained by the intervention, or by differing prognostic characteristics in subgroups that appear after randomisation.	**Yes**, if the study specified that subgroups were defined on the basis of characteristics at baseline.<br><br>**No**, if the study describes that the subgroups were defined according to characteristics measured after randomisation or did not describe when the subgroups were defined.
2. Was the subgroup variable a stratification factor at randomisation?	Credibility of subgroup difference would be increased if a subgroup variable was also used for stratification at randomisation (i.e. stratified randomisation).	**Yes**, if the randomisation included stratification based on the pre-specified subgroups variable.<br><br>No, if the study clearly reported information on stratification, but the subgroup variable of interest was not one of the stratification factors, or if no information was available regarding stratification.
3. Was the hypothesis specified a priori?	A subgroup analysis might be clearly planned before to test a hypothesis. This must be mentioned on the study protocol (registered or published) or primary trial, when appropriate. Post-hoc analyses are more susceptible to bias as well as spurious results and they should be viewed as hypothesis generating rather than hypothesis testing.	**Yes**, there needs to be a publicly available record (i.e. study protocol, registry, or primary trial) of the hypothesis that predates the subgroup analyses.<br><br>**No**, if the report specifically says the analyses were post-hoc, or no information

		reported regarding this aspect.
4. Was the subgroup analysis one of small number of subgroup hypotheses tested (≤5)?	The greater the number of hypotheses tested, the greater the number of interactions that will be discovered by chance, that is, the more likely it is to make a type I error (reject one of the null hypotheses even if all are actually true). A more appropriate analysis would account for the number of subgroups.	**Yes**, if the authors clearly specify up to 5 subgroup hypotheses.<br><br>**No**, if authors clearly specified more than 5 subgroup hypotheses, or if the study did not give this information.
**Analysis**		
5. Was the test of interaction significant (interaction P<0.05)?	Statistical tests of significance must be used to assess the likelihood that a given interaction might have arisen due to chance alone (the lower a P value is, the less likely it is that the interaction can be explained by chance).	**Yes**, if the study used any reliable statistical test to assess the subgroup interactions (e.g. regression models), and a P value lower than 0.05.<br><br>**No**, no reliable statistical test used, or P value higher than 0.05.
6. Was the significant interaction effect independent, if there were multiple significant interactions?	When testing multiple hypotheses in a single study, the analyses might yield more than one apparently significant interaction. These significant interactions might, however, be associated with each other, and thus explained by a common factor.	**Yes**, if the significant subgroup effect was not associated with other significant interactions, or if the subgroup effect was tested regarding its independence with other interaction effects (usually tested in multivariable regression that includes interaction terms).<br><br>**No**, if the subgroup effect was analysed only as part of a significant interaction effect.
**Context**		
7. Was the direction of subgroup effect correctly pre-specified?	A subgroup effect consistent with the pre-specified direction will increase the credibility of a subgroup analysis. Failure to specify the direction or even getting the wrong direction weakens	**Yes**, if the direction of subgroup effect was correctly specified a priori (e.g. study protocol, published

	the case for a real underlying subgroup effect	statistical analysis plan, trial registry).<br><br>**No**: if the authors fail to specify the direction or specify the wrong direction a priori.
8. Was the subgroup effect consistent with evidence from previous studies?	A hypothesis concerning differential response in a subgroup of patients may be generated by examination of data from a single study. The interaction becomes far more credible if it is also found in other similar studies. The extent to which a comprehensive scientific overview of the relevant literature finds an interaction to be consistently present is probably the best single index as to whether it should be believed. In other words, the replication of an interaction in independent, unbiased studies provides strong support for its believability.	**Yes**, if the study provides information that there was a consistent interaction found in other studies consistent with both the power of the comparisons and differences between studies that might influence results.<br><br>**No**, if the information provided by the study was not consistent across other studies, or if no information about other studies were reported.
9. Was the subgroup effect consistent across related outcomes?	The subgroup effect is more likely to be real if its effect manifest across all closely related outcomes. Studies must determine whether the subgroup effect existed among related outcomes.	**Yes**, if there was a consistent interaction of a subgroup across closely related outcomes within the study; that is, there was a consistency of the subgroup effect across the related outcomes.<br><br>**No**, if the study did not determine whether the subgroup effect exists across the related outcomes.
10. Was there indirect evidence to support the apparent subgroup effect (biological rationale, laboratory tests, animal studies)?	We are generally more ready to believe a hypothesised interaction if indirect evidence makes the interaction more plausible. That is, to the extent that a hypothesis is consistent with our current understanding of the biologic mechanisms of disease, we are more likely to believe it. Such understanding	**Yes**, if the study provides information that the consistent interaction of a subgroup is plausible to indirect evidence.<br><br>**No**, the significant interaction found was not

	comes from three types of indirect evidence: (i) from studies of different populations (including animal studies); (ii) from observations of interactions for similar interventions; and (iii) from results of studies of other related outcomes.	reasonable with indirect evidence, or no information reported regarding this issue.

Reference: Sun X, Briel M, Busse JW, et al. Credibility of claims of subgroup effects in randomised controlled trials: systematic review. *BMJ*. 2012;344:e1553.
