# Supplementary material for: Subgroup Analysis in Pulmonary Hypertension-Specific Therapy Clinical Trials: A Systematic Review
Source: J Pers Med. 2022 May 25;12(6):863. doi: 10.3390/jpm12060863 (PMC9224970; doi:10.3390/jpm12060863)
Supplement: Supplementary file 1 [file jpm-12-00863-s001.zip › Supplemental File S4.pdf]

Supplemental File S4. Articles excluded and the reason of exclusion

Articles excluded and the reason of exclusion	
**Reference**	**Exclusion criteria**
Badesch DB, Tapson VF, McGoon MD, Brundage BH, Rubin LJ, Wigley FM, Rich S, Barst RJ, Barrett PS, Kral KM, Jöbsis MM, Loyd JE, Murali S, Frost A, Girgis R, Bourge RC, Ralph DD, Elliott CG, Hill NS, Langleben D, Schilz RJ, McLaughlin VV, Robbins IM, Groves BM, Shapiro S, Medsger TA Jr. Continuous intravenous epoprostenol for pulmonary hypertension due to the scleroderma spectrum of disease. A randomized, controlled trial. Ann Intern Med. 2000 Mar 21;132(6):425-34. doi: 10.7326/0003-4819-132-6-200003210-00002. PMID: 10733441.	No subgroup analyses reported
Givertz MM, Colucci WS, LeJemtel TH, Gottlieb SS, Hare JM, Slawsky MT, Leier CV, Loh E, Nicklas JM, Lewis BE. Acute endothelin A receptor blockade causes selective pulmonary vasodilation in patients with chronic heart failure. Circulation. 2000 Jun 27;101(25):2922-7. doi: 10.1161/01.cir.101.25.2922. PMID: 10869264.	No subgroup analyses reported
Channick RN, Simonneau G, Sitbon O, Robbins IM, Frost A, Tapson VF, Badesch DB, Roux S, Rainisio M, Bodin F, Rubin LJ. Effects of the dual endothelin-receptor antagonist bosentan in patients with pulmonary hypertension: a randomised placebo-controlled study. Lancet. 2001 Oct 6;358(9288):1119-23. doi: 10.1016/S0140-6736(01)06250-X. PMID: 11597664.	No subgroup analyses reported
Gessler T, Schmehl T, Hoeper MM, Rose F, Ghofrani HA, Olschewski H, Grimminger F, Seeger W. Ultrasonic versus jet nebulization of iloprost in severe pulmonary hypertension. Eur Respir J. 2001 Jan;17(1):14-9. doi: 10.1183/09031936.01.17100140. PMID: 11307743.	No subgroup analyses reported
Ghofrani HA, Wiedemann R, Rose F, Schermuly RT, Olschewski H, Weissmann N, Gunther A, Walmrath D, Seeger W, Grimminger F. Sildenafil for treatment of lung fibrosis and pulmonary hypertension: a randomised controlled trial. Lancet. 2002 Sep 21;360(9337):895-900. doi: 10.1016/S0140-6736(02)11024-5. PMID: 12354470.	No subgroup analyses reported
Simonneau G, Barst RJ, Galie N, Naeije R, Rich S, Bourge RC, Keogh A, Oudiz R, Frost A, Blackburn SD, Crow JW, Rubin LJ; Treprostinil Study Group. Continuous subcutaneous infusion of treprostinil, a prostacyclin analogue, in patients with pulmonary arterial hypertension: a double-blind, randomized, placebo-controlled trial. Am J Respir Crit Care Med. 2002 Mar 15;165(6):800-4. doi: 10.1164/ajrccm.165.6.2106079. PMID: 11897647.	No subgroup analyses reported
Barst RJ, McGoon M, McLaughlin V, Tapson V, Rich S, Rubin L, Wasserman K, Oudiz R, Shapiro S, Robbins IM, Channick R, Badesch D, Rayburn BK, Flinchbaugh R, Sigman J, Arneson C, Jeffs R; Beraprost Study Group. Beraprost therapy for pulmonary arterial hypertension. J Am Coll Cardiol. 2003 Jun 18;41(12):2119-25. doi: 10.1016/s0735-1097(03)00463-7. Erratum in: J Am Coll Cardiol. 2003 Aug 6;42(3):591. PMID: 12821234.	No subgroup analyses reported

Galiè N, Hinderliter AL, Torbicki A, Fourme T, Simonneau G, Pulido T, Espinola-Zavaleta N, Rocchi G, Manes A, Frantz R, Kurzyna M, Nagueh SF, Barst R, Channick R, Dujardin K, Kronenberg A, Leconte I, Rainisio M, Rubin L. Effects of the oral endothelin-receptor antagonist bosentan on echocardiographic and doppler measures in patients with pulmonary arterial hypertension. J Am Coll Cardiol. 2003 Apr 16;41(8):1380-6. doi: 10.1016/s0735-1097(03)00121-9. PMID: 12706935.	Post-hoc analysis of a previously published RCT
Haché M, Denault A, Bélisle S, Robitaille D, Couture P, Sheridan P, Pellerin M, Babin D, Noël N, Guertin MC, Martineau R, Dupuis J. Inhaled epoprostenol (prostacyclin) and pulmonary hypertension before cardiac surgery. J Thorac Cardiovasc Surg. 2003 Mar;125(3):642-9. doi: 10.1067/mtc.2003.107. PMID: 12658208.	No subgroup analyses reported
Kanazawa H, Hirata K, Yoshikawa J. Effects of captopril administration on pulmonary haemodynamics and tissue oxygenation during exercise in ACE gene subtypes in patients with COPD: a preliminary study. Thorax. 2003 Jul;58(7):629-31. doi: 10.1136/thorax.58.7.629. PMID: 12832683; PMCID: PMC1746721.	No subgroup analyses reported
Vonbank K, Ziesche R, Higenbottam TW, Stiebellehner L, Petkov V, Schenk P, Germann P, Block LH. Controlled prospective randomised trial on the effects on pulmonary haemodynamics of the ambulatory long term use of nitric oxide and oxygen in patients with severe COPD. Thorax. 2003 Apr;58(4):289-93. doi: 10.1136/thorax.58.4.289. PMID: 12668787; PMCID: PMC1746623.	No subgroup analyses reported
Barst RJ, Langleben D, Frost A, Horn EM, Oudiz R, Shapiro S, McLaughlin V, Hill N, Tapson VF, Robbins IM, Zwicke D, Duncan B, Dixon RA, Frumkin LR; STRIDE-1 Study Group. Sitaxsentan therapy for pulmonary arterial hypertension. Am J Respir Crit Care Med. 2004 Feb 15;169(4):441-7. doi: 10.1164/rccm.200307-957OC. Epub 2003 Nov 20. PMID: 14630619.	No subgroup analyses reported
Ghofrani HA, Voswinckel R, Reichenberger F, Olschewski H, Haredza P, Karadaş B, Schermuly RT, Weissmann N, Seeger W, Grimminger F. Differences in hemodynamic and oxygenation responses to three different phosphodiesterase-5 inhibitors in patients with pulmonary arterial hypertension: a randomized prospective study. J Am Coll Cardiol. 2004 Oct 6;44(7):1488-96. doi: 10.1016/j.jacc.2004.06.060. PMID: 15464333.	No subgroup analyses reported
Goldsmith DR, Wagstaff AJ. Inhaled iloprost: in primary pulmonary hypertension. Drugs. 2004;64(7):763-73; discussion 774-5. doi: 10.2165/00003495-200464070-00009. PMID: 15025551.	The article did not report results from a controlled randomized trial
Humbert M, Barst RJ, Robbins IM, Channick RN, Galiè N, Boonstra A, Rubin LJ, Horn EM, Manes A, Simonneau G. Combination of bosentan with epoprostenol in pulmonary arterial hypertension: BREATHE-2. Eur Respir J. 2004 Sep;24(3):353-9. doi: 10.1183/09031936.04.00028404. PMID: 15358690.	No subgroup analyses reported

Langleben D, Brock T, Dixon R, Barst R; STRIDE-1 study group. STRIDE 1: effects of the selective ET(A) receptor antagonist, sitaxsentan sodium, in a patient population with pulmonary arterial hypertension that meets traditional inclusion criteria of previous pulmonary arterial hypertension trials. J Cardiovasc Pharmacol. 2004 Nov;44 Suppl 1:S80-4. doi: 10.1097/01.fjc.0000166207.74178.d0. PMID: 15838366.	Post-hoc analysis of a previously published RCT
Oudiz RJ, Schilz RJ, Barst RJ, Galié N, Rich S, Rubin LJ, Simonneau G; Treprostinil Study Group. Treprostinil, a prostacyclin analogue, in pulmonary arterial hypertension associated with connective tissue disease. Chest. 2004 Aug;126(2):420-7. doi: 10.1378/chest.126.2.420. PMID: 15302727.	The article did not report results from a controlled randomized trial
Sastry BK, Narasimhan C, Reddy NK, Raju BS. Clinical efficacy of sildenafil in primary pulmonary hypertension: a randomized, placebo-controlled, double-blind, crossover study. J Am Coll Cardiol. 2004 Apr 7;43(7):1149-53. doi: 10.1016/j.jacc.2003.10.056. PMID: 15063421.	No subgroup analyses reported
Aldashev AA, Kojonazarov BK, Amatov TA, Sooronbaev TM, Mirrakhimov MM, Morrell NW, Wharton J, Wilkins MR. Phosphodiesterase type 5 and high altitude pulmonary hypertension. Thorax. 2005 Aug;60(8):683-7. doi: 10.1136/thx.2005.041954. PMID: 16061711; PMCID: PMC1747498.	No subgroup analyses reported
Fattouch K, Sbraga F, Bianco G, Speziale G, Gucciardo M, Sampognaro R, Ruvolo G. Inhaled prostacyclin, nitric oxide, and nitroprusside in pulmonary hypertension after mitral valve replacement. J Card Surg. 2005 Mar-Apr;20(2):171-6. doi: 10.1111/j.0886-0440.2005.200383w.x. PMID: 15725144.	No subgroup analyses reported
Genovese MC, Chakravarty EF, Boyle DL, Tutuncu Z, Thorburn CM, Halilhodzic M, Kroll S, Baughman J, Stewart S, Kavanaugh A. A randomized, blinded, parallel group, placebo controlled pilot study evaluating the effect of PVAC treatment in patients with diffuse systemic sclerosis. J Rheumatol. 2005 Dec;32(12):2345-50. PMID: 16331761.	The article was not available
Hoeper MM, Kramm T, Wilkens H, Schulze C, Schäfers HJ, Welte T, Mayer E. Bosentan therapy for inoperable chronic thromboembolic pulmonary hypertension. Chest. 2005 Oct;128(4):2363-7. doi: 10.1378/chest.128.4.2363. PMID: 16236895.	No subgroup analyses reported
Kramm T, Eberle B, Guth S, Mayer E. Inhaled iloprost to control residual pulmonary hypertension following pulmonary endarterectomy. Eur J Cardiothorac Surg. 2005 Dec;28(6):882-8. doi: 10.1016/j.ejcts.2005.09.007. Epub 2005 Oct 19. PMID: 16242948.	No subgroup analyses reported
McLaughlin VV, Sitbon O, Badesch DB, Barst RJ, Black C, Galiè N, Rainisio M, Simonneau G, Rubin LJ. Survival with first-line bosentan in patients with primary pulmonary hypertension. Eur Respir J. 2005 Feb;25(2):244-9. doi: 10.1183/09031936.05.00054804. Erratum in: Eur Respir J. 2005 May;25(5):942. PMID: 15684287.	No subgroup analyses reported

Morrell NW, Higham MA, Phillips PG, Shakur BH, Robinson PJ, Beddoes RJ. Pilot study of losartan for pulmonary hypertension in chronic obstructive pulmonary disease. Respir Res. 2005 Aug 1;6(1):88. doi: 10.1186/1465-9921-6-88. PMID: 16060962; PMCID: PMC1198258.	No subgroup analyses reported
Ocal A, Kiriş I, Erdinç M, Peker O, Yavuz T, Ibrişim E. Efficiency of prostacyclin in the treatment of protamine-mediated right ventricular failure and acute pulmonary hypertension. Tohoku J Exp Med. 2005 Sep;207(1):51-8. doi: 10.1620/tjem.207.51. PMID: 16082155.	No subgroup analyses reported
Radovancevic B, Vrtovec B, Thomas CD, Croitoru M, Myers TJ, Radovancevic R, Khan T, Massin EK, Frazier OH. Nitric oxide versus prostaglandin E1 for reduction of pulmonary hypertension in heart transplant candidates. J Heart Lung Transplant. 2005 Jun;24(6):690-5. doi: 10.1016/j.healun.2004.04.016. PMID: 15949728.	No subgroup analyses reported
Seyfarth HJ, Pankau H, Hammerschmidt S, Schauer J, Wirtz H, Winkler J. Bosentan improves exercise tolerance and Tei index in patients with pulmonary hypertension and prostanoid therapy. Chest. 2005 Aug;128(2):709-13. doi: 10.1378/chest.128.2.709. PMID: 16100158.	No subgroup analyses reported
Shen J, He B, Wang B. Effects of lipo-prostaglandin E1 on pulmonary hemodynamics and clinical outcomes in patients with pulmonary arterial hypertension. Chest. 2005 Aug;128(2):714-9. doi: 10.1378/chest.128.2.714. PMID: 16100159.	No subgroup analyses reported
Wilkins MR, Paul GA, Strange JW, Tunariu N, Gin-Sing W, Banya WA, Westwood MA, Stefanidis A, Ng LL, Pennell DJ, Mohiaddin RH, Nihoyannopoulos P, Gibbs JS. Sildenafil versus Endothelin Receptor Antagonist for Pulmonary Hypertension (SERAPH) study. Am J Respir Crit Care Med. 2005 Jun 1;171(11):1292-7. doi: 10.1164/rccm.200410-1411OC. Epub 2005 Mar 4. PMID: 15750042.	No subgroup analyses reported
Fattouch K, Sbraga F, Sampognaro R, Bianco G, Gucciardo M, Lavalle C, Vizza CD, Fedele F, Ruvolo G. Treatment of pulmonary hypertension in patients undergoing cardiac surgery with cardiopulmonary bypass: a randomized, prospective, double-blind study. J Cardiovasc Med (Hagerstown). 2006 Feb;7(2):119-23. doi: 10.2459/01.JCM.0000203850.97890.fe. PMID: 16645371.	No subgroup analyses reported
Galiè N, Beghetti M, Gatzoulis MA, Granton J, Berger RM, Lauer A, Chiossi E, Landzberg M; Bosentan Randomized Trial of Endothelin Antagonist Therapy-5 (BREATHE-5) Investigators. Bosentan therapy in patients with Eisenmenger syndrome: a multicenter, double-blind, randomized, placebo-controlled study. Circulation. 2006 Jul 4;114(1):48-54. doi: 10.1161/CIRCULATIONAHA.106.630715. Epub 2006 Jun 26. PMID: 16801459.	No subgroup analyses reported
McLaughlin VV, Oudiz RJ, Frost A, Tapson VF, Murali S, Channick RN, Badesch DB, Barst RJ, Hsu HH, Rubin LJ. Randomized study of adding inhaled iloprost to existing bosentan in pulmonary arterial hypertension.	No subgroup analyses reported

Am J Respir Crit Care Med. 2006 Dec 1;174(11):1257-63. doi: 10.1164/rccm.200603-358OC. Epub 2006 Aug 31. PMID: 16946127.	
Singh TP, Rohit M, Grover A, Malhotra S, Vijayvergiya R. A randomized, placebo-controlled, double-blind, crossover study to evaluate the efficacy of oral sildenafil therapy in severe pulmonary artery hypertension. Am Heart J. 2006 Apr;151(4):851.e1-5. doi: 10.1016/j.ahj.2005.09.006. PMID: 16569546.	Subgroup analyses missing data
Voswinckel R, Enke B, Reichenberger F, Kohstall M, Kreckel A, Krick S, Gall H, Gessler T, Schmehl T, Ghofrani HA, Schermuly RT, Grimminger F, Rubin LJ, Seeger W, Olschewski H. Favorable effects of inhaled treprostinil in severe pulmonary hypertension: results from randomized controlled pilot studies. J Am Coll Cardiol. 2006 Oct 17;48(8):1672-81. doi: 10.1016/j.jacc.2006.06.062. Epub 2006 Sep 26. PMID: 17045906.	No subgroup analyses reported
Barst RJ, Langleben D, Badesch D, Frost A, Lawrence EC, Shapiro S, Naeije R, Galie N; STRIDE-2 Study Group. Treatment of pulmonary arterial hypertension with the selective endothelin-A receptor antagonist sitaxsentan. J Am Coll Cardiol. 2006 May 16;47(10):2049-56. doi: 10.1016/j.jacc.2006.01.057. Epub 2006 Apr 24. PMID: 16697324.	No subgroup analyses reported
Hoeper MM, Leuchte H, Halank M, Wilkens H, Meyer FJ, Seyfarth HJ, Wensel R, Ripken F, Bremer H, Kluge S, Hoeffken G, Behr J. Combining inhaled iloprost with bosentan in patients with idiopathic pulmonary arterial hypertension. Eur Respir J. 2006 Oct;28(4):691-4. doi: 10.1183/09031936.06.00057906. PMID: 17012628.	No subgroup analyses reported
Barst RJ, Galie N, Naeije R, Simonneau G, Jeffs R, Arneson C, Rubin LJ. Long-term outcome in pulmonary arterial hypertension patients treated with subcutaneous treprostinil. Eur Respir J. 2006 Dec;28(6):1195-203. doi: 10.1183/09031936.06.00044406. Epub 2006 Aug 9. PMID: 16899485.	No subgroup analyses reported
Ludwik B, Lewczuk J, Piszko P, Nowak M, Wrabec K. Roczna, kontrolowana ocena stosowania doksazosyny u chorych na nadciśnienie płucne na tie przewlekłe j obturacyjnej choroby płuc [One-year controlled therapy with doxazosin in patients with pulmonary hypertension due to chronic obstructive pulmonary disease]. Pol Merkur Lekarski. 2006 May;20(119):527-30. Polish. PMID: 16875154.	The article was not available
McLaughlin VV. Survival in patients with pulmonary arterial hypertension treated with first-line bosentan. Eur J Clin Invest. 2006 Sep;36 Suppl 3:10-5. doi: 10.1111/j.1365-2362.2006.01688.x. PMID: 16919005.	No subgroup analyses reported
But AK, Ozgul U, Erdil F, Gulhas N, Toprak HI, Durmus M, Ersoy MO. The effects of pre-operative dexmedetomidine infusion on hemodynamics in patients with pulmonary hypertension undergoing mitral valve replacement surgery. Acta Anaesthesiol Scand. 2006 Nov;50(10):1207-12. doi: 10.1111/j.1399-6576.2006.01136.x. Epub 2006 Sep 15. PMID: 16978159.	No subgroup analyses reported
Shim JK, Choi YS, Oh YJ, Kim DH, Hong YW, Kwak YL. Effect of oral sildenafil	No subgroup analyses

citrate on intraoperative hemodynamics in patients with pulmonary hypertension undergoing valvular heart surgery. J Thorac Cardiovasc Surg. 2006 Dec;132(6):1420-5. doi: 10.1016/j.jtcvs.2006.08.035. PMID: 17140970.	reported
Yurtseven N, Karaca P, Uysal G, Ozkul V, Cimen S, Tuygun AK, Yuksek A, Canik S. A comparison of the acute hemodynamic effects of inhaled nitroglycerin and iloprost in patients with pulmonary hypertension undergoing mitral valve surgery. Ann Thorac Cardiovasc Surg. 2006 Oct;12(5):319-23. PMID: 17095973.	The article was not available
Yi S, Ran L, Gu XH. [Effects of pulmonary arterial perfusion with shenqi fuzheng injection on lung injury during cardiopulmonary bypass]. Zhongguo Zhong Xi Yi Jie He Za Zhi. 2006 Oct;26(10):938-41. Chinese. PMID: 17121051.	The article was written in a language other than english/spanish/french
Badesch DB, Hill NS, Burgess G, Rubin LJ, Barst RJ, Galiè N, Simonneau G; SUPER Study Group. Sildenafil for pulmonary arterial hypertension associated with connective tissue disease. J Rheumatol. 2007 Dec;34(12):2417-22. Epub 2007 Nov 1. PMID: 17985403.	Post-hoc analysis of a previously published RCT
Benza RL, Mehta S, Keogh A, Lawrence EC, Oudiz RJ, Barst RJ. Sitaxsentan treatment for patients with pulmonary arterial hypertension discontinuing bosentan. J Heart Lung Transplant. 2007 Jan;26(1):63-9. doi: 10.1016/j.healun.2006.10.019. PMID: 17234519.	No subgroup analyses reported
Lewis GD, Shah R, Shahzad K, Camuso JM, Pappagianopoulos PP, Hung J, Tawakol A, Gerszten RE, Systrom DM, Bloch KD, Semigran MJ. Sildenafil improves exercise capacity and quality of life in patients with systolic heart failure and secondary pulmonary hypertension. Circulation. 2007 Oct 2;116(14):1555-62. doi: 10.1161/CIRCULATIONAHA.107.716373. Epub 2007 Sep 4. PMID: 17785618.	No subgroup analyses reported
Bharani A, Patel A, Saraf J, Jain A, Mehrotra S, Lunia B. Efficacy and safety of PDE-5 inhibitor tadalafil in pulmonary arterial hypertension. Indian Heart J. 2007 Jul-Aug;59(4):323-8. PMID: 19126937.	The article was not available
Rubenfire M, McLaughlin VV, Allen RP, Elliott G, Park MH, Wade M, Schilz R. Transition from IV epoprostenol to subcutaneous treprostinil in pulmonary arterial hypertension: a controlled trial. Chest. 2007 Sep;132(3):757-63. doi: 10.1378/chest.06-2118. Epub 2007 Mar 30. Erratum in: Chest. 2007 Nov;132(5):1721. PMID: 17400684.	No subgroup analyses reported
Sfikakis PP, Papamichael C, Stamatelopoulos KS, Tousoulis D, Fragiadaki KG, Katsichti P, Stefanadis C, Mavrikakis M. Improvement of vascular endothelial function using the oral endothelin receptor antagonist bosentan in patients with systemic sclerosis. Arthritis Rheum. 2007 Jun;56(6):1985-93. doi: 10.1002/art.22634. PMID: 17530638.	The trial was not design to include pulmonary hypertension patients
Oztekin I, Yazici S, Oztekin DS, Goksel O, Issever H, Canik S. Effects of low-dose milrinone on weaning from cardiopulmonary bypass and after in	No subgroup analyses reported

patients with mitral stenosis and pulmonary hypertension. Yakugaku Zasshi. 2007 Feb;127(2):375-83. doi: 10.1248/yakushi.127.375. PMID: 17268158.	
Kaluski E, Cotter G, Leitman M, Milo-Cotter O, Krakover R, Kobrin I, Moriconi T, Rainisio M, Caspi A, Reizin L, Zimlichman R, Vered Z. Clinical and hemodynamic effects of bosentan dose optimization in symptomatic heart failure patients with severe systolic dysfunction, associated with secondary pulmonary hypertension--a multi-center randomized study. Cardiology. 2008;109(4):273-80. doi: 10.1159/000107791. Epub 2007 Sep 17. PMID: 17873492.	No subgroup analyses reported
Skoro-Sajer N, Lang IM, Harja E, Kneussl MP, Sing WG, Gibbs SJ. A clinical comparison of slow- and rapid-escalation treprostinil dosing regimens in patients with pulmonary hypertension. Clin Pharmacokinet. 2008;47(9):611-8. doi: 10.2165/00003088-200847090-00004. PMID: 18698881.	No subgroup analyses reported
Suntharalingam J, Treacy CM, Doughty NJ, Goldsmith K, Soon E, Toshner MR, Sheares KK, Hughes R, Morrell NW, Pepke-Zaba J. Long-term use of sildenafil in inoperable chronic thromboembolic pulmonary hypertension. Chest. 2008 Aug;134(2):229-236. doi: 10.1378/chest.07-2681. Epub 2008 Feb 8. PMID: 18263674.	No subgroup analyses reported
von Dossow V, Spies C, Schenk H, Schlesinger S, von Heymann C. Secondary pulmonary hypertension: haemodynamic effects of torasemide versus furosemide. Clin Drug Investig. 2008;28(1):17-26. doi: 10.2165/00044011-200828010-00003. PMID: 18081357.	No subgroup analyses reported
Behling A, Rohde LE, Colombo FC, Goldraich LA, Stein R, Clausell N. Effects of 5'-phosphodiesterase four-week long inhibition with sildenafil in patients with chronic heart failure: a double-blind, placebo-controlled clinical trial. J Card Fail. 2008 Apr;14(3):189-97. doi: 10.1016/j.cardfail.2007.11.006. PMID: 18381181.	No subgroup analyses reported
Stolz D, Rasch H, Linka A, Di Valentino M, Meyer A, Brutsche M, Tamm M. A randomised, controlled trial of bosentan in severe COPD. Eur Respir J. 2008 Sep;32(3):619-28. doi: 10.1183/09031936.00011308. Epub 2008 Apr 30. PMID: 18448495.	No subgroup analyses reported
Pepke-Zaba J, Gilbert C, Collings L, Brown MC. Sildenafil improves health-related quality of life in patients with pulmonary arterial hypertension. Chest. 2008 Jan;133(1):183-9. doi: 10.1378/chest.07-0592. PMID: 18187744.	No subgroup analyses reported
Galiè N, Olschewski H, Oudiz RJ, Torres F, Frost A, Ghofrani HA, Badesch DB, McGoon MD, McLaughlin VV, Roecker EB, Gerber MJ, Dufton C, Wiens BL, Rubin LJ; Ambrisentan in Pulmonary Arterial Hypertension, Randomized, Double-Blind, Placebo-Controlled, Multicenter, Efficacy Studies (ARIES) Group. Ambrisentan for the treatment of pulmonary arterial hypertension: results of the ambrisentan in pulmonary arterial hypertension, randomized, double-blind, placebo-controlled, multicenter, efficacy (ARIES) study 1 and	No subgroup analyses reported

2. Circulation. 2008 Jun 10;117(23):3010-9. doi: 10.1161/CIRCULATIONAHA.107.742510. Epub 2008 May 27. PMID: 18506008.	
Cai J, Su Z, Shi Z, Zhou Y, Xu Z, Liu J, Chen L, Xu Z, Yu X, Ding W, Yang Y. Nitric oxide in conjunction with milrinone better stabilized pulmonary hemodynamics after Fontan procedure. Artif Organs. 2008 Nov;32(11):864-9. doi: 10.1111/j.1525-1594.2008.00643.x. PMID: 18959679.	No subgroup analyses reported
Winterhalter M, Simon A, Fischer S, Rahe-Meyer N, Chamtzidou N, Hecker H, Zuk J, Piepenbrock S, Strüber M. Comparison of inhaled iloprost and nitric oxide in patients with pulmonary hypertension during weaning from cardiopulmonary bypass in cardiac surgery: a prospective randomized trial. J Cardiothorac Vasc Anesth. 2008 Jun;22(3):406-13. doi: 10.1053/j.jvca.2007.10.015. Epub 2008 Jan 22. PMID: 18503929.	No subgroup analyses reported
Kleber FX, Bollmann T, Borst MM, Costard-Jäckle A, Ewert R, Kivikko M, Petterson T, Pohjanjousi P, Sonntag S, Wikström G. Repetitive dosing of intravenous levosimendan improves pulmonary hemodynamics in patients with pulmonary hypertension: results of a pilot study. J Clin Pharmacol. 2009 Jan;49(1):109-15. doi: 10.1177/0091270008325150. Epub 2008 Nov 3. PMID: 18981240.	No subgroup analyses reported
Baughman RP, Judson MA, Lower EE, Highland K, Kwon S, Craft N, Engel PJ. Inhaled iloprost for sarcoidosis associated pulmonary hypertension. Sarcoidosis Vasc Diffuse Lung Dis. 2009 Jul;26(2):110-20. PMID: 20560291.	The article was not available
Khan TA, Schnickel G, Ross D, Bastani S, Laks H, Esmailian F, Marelli D, Beygui R, Shemin R, Watson L, Vartapetian I, Ardehali A. A prospective, randomized, crossover pilot study of inhaled nitric oxide versus inhaled prostacyclin in heart transplant and lung transplant recipients. J Thorac Cardiovasc Surg. 2009 Dec;138(6):1417-24. doi: 10.1016/j.jtcvs.2009.04.063. PMID: 19931670.	No subgroup analyses reported
Seheult RD, Ruh K, Foster GP, Anholm JD. Prophylactic bosentan does not improve exercise capacity or lower pulmonary artery systolic pressure at high altitude. Respir Physiol Neurobiol. 2009 Feb 28;165(2-3):123-30. doi: 10.1016/j.resp.2008.10.005. Epub 2008 Oct 11. PMID: 18977464.	No subgroup analyses reported
Oudiz RJ, Galiè N, Olschewski H, Torres F, Frost A, Ghofrani HA, Badesch DB, McGoon MD, McLaughlin VV, Roecker EB, Harrison BC, Despain D, Dufton C, Rubin LJ; ARIES Study Group. Long-term ambrisentan therapy for the treatment of pulmonary arterial hypertension. J Am Coll Cardiol. 2009 Nov 17;54(21):1971-81. doi: 10.1016/j.jacc.2009.07.033. PMID: 19909879.	No subgroup analyses reported
Valerio G, Bracciale P, Grazia D'Agostino A. Effect of bosentan upon pulmonary hypertension in chronic obstructive pulmonary disease. Ther Adv Respir Dis. 2009 Feb;3(1):15-21. doi: 10.1177/1753465808103499. PMID: 19293199.	No subgroup analyses reported
Pepke-Zaba J, Beardsworth A, Chan M, Angalakuditi M. Tadalafil therapy	No subgroup analyses

and health-related quality of life in pulmonary arterial hypertension. Curr Med Res Opin. 2009 Oct;25(10):2479-85. doi: 10.1185/03007990903210066. PMID: 19686085.	reported
Wang H, Gong M, Zhou B, Dai A. Comparison of inhaled and intravenous milrinone in patients with pulmonary hypertension undergoing mitral valve surgery. Adv Ther. 2009 Apr;26(4):462-8. doi: 10.1007/s12325-009-0019-4. Epub 2009 Apr 16. PMID: 19381522.	No subgroup analyses reported
Botha P, Parry G, Dark JH, Macgowan GA. Acute hemodynamic effects of intravenous sildenafil citrate in congestive heart failure: comparison of phosphodiesterase type-3 and -5 inhibition. J Heart Lung Transplant. 2009 Jul;28(7):676-82. doi: 10.1016/j.healun.2009.04.013. Epub 2009 May 13. PMID: 19560695.	No subgroup analyses reported
Ghofrani HA, Hoeper MM, Halank M, Meyer FJ, Staehler G, Behr J, Ewert R, Weimann G, Grimminger F. Riociguat for chronic thromboembolic pulmonary hypertension and pulmonary arterial hypertension: a phase II study. Eur Respir J. 2010 Oct;36(4):792-9. doi: 10.1183/09031936.00182909. Epub 2010 Jun 7. PMID: 20530034.	The article did not report results from a controlled randomized trial
Reesink HJ, Surie S, Kloek JJ, Tan HL, Tepaske R, Fedullo PF, Bresser P. Bosentan as a bridge to pulmonary endarterectomy for chronic thromboembolic pulmonary hypertension. J Thorac Cardiovasc Surg. 2010 Jan;139(1):85-91. doi: 10.1016/j.jtcvs.2009.03.053. Epub 2009 May 31. PMID: 19660388.	No subgroup analyses reported
Bozbaş SS, Bozbaş H, Atar A, Ulubay G, Oner Eyüboğlu F. Comparative effects of losartan and nifedipine therapy on exercise capacity, Doppler echocardiographic parameters and endothelin levels in patients with secondary pulmonary hypertension. Anadolu Kardiyol Derg. 2010 Feb;10(1):43-9. PMID: 20150004.	The article was not available
McLaughlin VV, Benza RL, Rubin LJ, Channick RN, Voswinckel R, Tapson VF, Robbins IM, Olschewski H, Rubenfire M, Seeger W. Addition of inhaled treprostinil to oral therapy for pulmonary arterial hypertension: a randomized controlled clinical trial. J Am Coll Cardiol. 2010 May 4;55(18):1915-22. doi: 10.1016/j.jacc.2010.01.027. PMID: 20430262.	No subgroup analyses reported
Blanco I, Gimeno E, Munoz PA, Pizarro S, Gistau C, Rodriguez-Roisin R, Roca J, Barberà JA. Hemodynamic and gas exchange effects of sildenafil in patients with chronic obstructive pulmonary disease and pulmonary hypertension. Am J Respir Crit Care Med. 2010 Feb 1;181(3):270-8. doi: 10.1164/rccm.200907-0988OC. Epub 2009 Oct 29. PMID: 19875684.	No subgroup analyses reported
Hiremath J, Thanikachalam S, Parikh K, Shanmugasundaram S, Bangera S, Shapiro L, Pott GB, Vnencak-Jones CL, Arneson C, Wade M, White RJ; TRUST Study Group. Exercise improvement and plasma biomarker changes with intravenous treprostinil therapy for pulmonary arterial hypertension: a placebo-controlled trial. J Heart Lung Transplant. 2010 Feb;29(2):137-49. doi: 10.1016/j.healun.2009.09.005. PMID: 20022264.	No subgroup analyses reported

Toshner MR, Gopalan D, Suntharalingam J, Treacy C, Soon E, Sheares KK, Morrell NW, Screaton N, Pepke-Zaba J. Pulmonary arterial size and response to sildenafil in chronic thromboembolic pulmonary hypertension. J Heart Lung Transplant. 2010 Jun;29(6):610-5. doi: 10.1016/j.healun.2009.12.014. Epub 2010 Mar 15. PMID: 20227301; PMCID: PMC2954311.	No subgroup analyses reported
Seibold JR, Denton CP, Furst DE, Guillevin L, Rubin LJ, Wells A, Matucci Cerinic M, Riemekasten G, Emery P, Chadha-Boreham H, Charef P, Roux S, Black CM. Randomized, prospective, placebo-controlled trial of bosentan in interstitial lung disease secondary to systemic sclerosis. Arthritis Rheum. 2010 Jul;62(7):2101-8. doi: 10.1002/art.27466. Erratum in: Arthritis Rheum. 2010 Oct;62(10):3005. PMID: 20506355.	No subgroup analyses reported
Ghofrani HA, Morrell NW, Hoeper MM, Olschewski H, Peacock AJ, Barst RJ, Shapiro S, Golpon H, Toshner M, Grimminger F, Pascoe S. Imatinib in pulmonary arterial hypertension patients with inadequate response to established therapy. Am J Respir Crit Care Med. 2010 Nov 1;182(9):1171-7. doi: 10.1164/rccm.201001-0123OC. Epub 2010 Jun 25. PMID: 20581169; PMCID: PMC3001259.	The article did not report on pulmonary hypertension specific-therapy
Kim SY, Shim JK, Shim YH, Hong SW, Choi KH, Kwak YL. Sildenafil and beraprost combination therapy in patients with pulmonary hypertension undergoing valvular heart surgery. J Heart Valve Dis. 2010 May;19(3):333-40. PMID: 20583396.	The article was not available
Barst RJ, Mubarak KK, Machado RF, Ataga KI, Benza RL, Castro O, Naeije R, Sood N, Swerdlow PS, Hildesheim M, Gladwin MT; ASSET study group*. Exercise capacity and haemodynamics in patients with sickle cell disease with pulmonary hypertension treated with bosentan: results of the ASSET studies. Br J Haematol. 2010 May;149(3):426-35. doi: 10.1111/j.1365-2141.2010.08097.x. Epub 2010 Feb 17. PMID: 20175775; PMCID: PMC2914575.	No subgroup analyses reported
Fernandes JL, Sampaio RO, Brandão CM, Accorsi TA, Cardoso LF, Spina GS, Tarasoutchi F, Pomerantzeff P, Auler JO Jr, Grinberg M. Comparison of inhaled nitric oxide versus oxygen on hemodynamics in patients with mitral stenosis and severe pulmonary hypertension after mitral valve surgery. Am J Cardiol. 2011 Apr 1;107(7):1040-5. doi: 10.1016/j.amjcard.2010.11.030. Epub 2011 Feb 4. PMID: 21296315.	No subgroup analyses reported
Jing ZC, Yu ZX, Shen JY, Wu BX, Xu KF, Zhu XY, Pan L, Zhang ZL, Liu XQ, Zhang YS, Jiang X, Galiè N; Efficacy and Safety of Vardenafil in the Treatment of Pulmonary Arterial Hypertension (EVALUATION) Study Group. Vardenafil in pulmonary arterial hypertension: a randomized, double-blind, placebo-controlled study. Am J Respir Crit Care Med. 2011 Jun 15;183(12):1723-9. doi: 10.1164/rccm.201101-0093OC. Epub 2011 Mar 11. PMID: 21471085.	No subgroup analyses reported
Guazzi M, Vicenzi M, Arena R, Guazzi MD. Pulmonary hypertension in heart failure with preserved ejection fraction: a target of phosphodiesterase-5	No subgroup analyses reported

inhibition in a 1-year study. Circulation. 2011 Jul 12;124(2):164-74. doi: 10.1161/CIRCULATIONAHA.110.983866. Epub 2011 Jun 27. PMID: 21709061.	
Benza RL, Seeger W, McLaughlin VV, Channick RN, Voswinckel R, Tapson VF, Robbins IM, Olschewski H, Rubin LJ. Long-term effects of inhaled treprostinil in patients with pulmonary arterial hypertension: the Treprostinil Sodium Inhalation Used in the Management of Pulmonary Arterial Hypertension (TRIUMPH) study open-label extension. J Heart Lung Transplant. 2011 Dec;30(12):1327-33. doi: 10.1016/j.healun.2011.08.019. PMID: 22055098.	No subgroup analyses reported
Bates MG, Thompson AA, Baillie JK, Sutherland AI, Irving JB, Hirani N, Webb DJ. Sildenafil citrate for the prevention of high altitude hypoxic pulmonary hypertension: double blind, randomized, placebo-controlled trial. High Alt Med Biol. 2011 Fall;12(3):207-14. doi: 10.1089/ham.2011.0007. PMID: 21962063.	Patients were not treated for pulmonary hypertension
Zhang DZ, Zhu XY, Meng J, Xue HM, Sheng XT, Han XM, Cui CS, Wang QG, Zhang P. Acute hemodynamic responses to adenosine and iloprost in patients with congenital heart defects and severe pulmonary arterial hypertension. Int J Cardiol. 2011 Mar 17;147(3):433-7. doi: 10.1016/j.ijcard.2010.04.093. PMID: 20537740.	No subgroup analyses reported
Rubin LJ, Badesch DB, Fleming TR, Galiè N, Simonneau G, Ghofrani HA, Oakes M, Layton G, Serdarevic-Pehar M, McLaughlin VV, Barst RJ; SUPER-2 Study Group. Long-term treatment with sildenafil citrate in pulmonary arterial hypertension: the SUPER-2 study. Chest. 2011 Nov;140(5):1274-1283. doi: 10.1378/chest.10-0969. Epub 2011 May 5. PMID: 21546436.	No subgroup analyses reported
Badesch DB, Feldman J, Keogh A, Mathier MA, Oudiz RJ, Shapiro S, Farber HW, McGoon M, Frost A, Allard M, Despain D, Dufton C, Rubin LJ; ARIES-3 Study Group. ARIES-3: ambrisentan therapy in a diverse population of patients with pulmonary hypertension. Cardiovasc Ther. 2012 Apr;30(2):93-9. doi: 10.1111/j.1755-5922.2011.00279.x. Epub 2011 Jun 20. PMID: 21884013.	The article did not report results from a controlled randomized trial
Boeck L, Tamm M, Grendelmeier P, Stolz D. Acute effects of aerosolized iloprost in COPD related pulmonary hypertension - a randomized controlled crossover trial. PLoS One. 2012;7(12):e52248. doi: 10.1371/journal.pone.0052248. Epub 2012 Dec 27. PMID: 23300624; PMCID: PMC3531427.	No subgroup analyses reported
Ghio S, Bonderman D, Felix SB, Ghofrani HA, Michelakis ED, Mitrovic V, Oudiz RJ, Frey R, Roessig L, Semigran MJ. Left ventricular systolic dysfunction associated with pulmonary hypertension riociguat trial (LEPHT): rationale and design. Eur J Heart Fail. 2012 Aug;14(8):946-53. doi: 10.1093/eurjhf/hfs071. Epub 2012 Jun 20. PMID: 22719060.	Clinical Trial protocol
Guazzi M, Vicenzi M, Arena R. Phosphodiesterase 5 inhibition with sildenafil reverses exercise oscillatory breathing in chronic heart failure: a long-term	No subgroup analyses reported

cardiopulmonary exercise testing placebo-controlled study. Eur J Heart Fail. 2012 Jan;14(1):82-90. doi: 10.1093/eurjhf/hfr147. Epub 2011 Nov 7. PMID: 22067410.	
Matamis D, Pampori S, Papathanasiou A, Papakonstantinou P, Tsagourias M, Galiatsou E, Koulouras V, Nakos G. Inhaled NO and sildenafil combination in cardiac surgery patients with out-of-proportion pulmonary hypertension: acute effects on postoperative gas exchange and hemodynamics. Circ Heart Fail. 2012 Jan;5(1):47-53. doi: 10.1161/CIRCHEARTFAILURE.111.963314. Epub 2011 Nov 4. PMID: 22057829.	No subgroup analyses reported
Simonneau G, Torbicki A, Hoeper MM, Delcroix M, Karlócai K, Galiè N, Degano B, Bonderman D, Kurzyna M, Efficace M, Giorgino R, Lang IM. Selexipag: an oral, selective prostacyclin receptor agonist for the treatment of pulmonary arterial hypertension. Eur Respir J. 2012 Oct;40(4):874-80. doi: 10.1183/09031936.00137511. Epub 2012 Feb 23. PMID: 22362844.	No subgroup analyses reported
Lederer DJ, Bartels MN, Schluger NW, Brogan F, Jellen P, Thomashow BM, Kawut SM. Sildenafil for chronic obstructive pulmonary disease: a randomized crossover trial. COPD. 2012 Jun;9(3):268-75. doi: 10.3109/15412555.2011.651180. Epub 2012 Feb 23. PMID: 22360383; PMCID: PMC4904720.	Patients were not treated for pulmonary hypertension
Tapson VF, Torres F, Kermeen F, Keogh AM, Allen RP, Frantz RP, Badesch DB, Frost AE, Shapiro SM, Laliberte K, Sigman J, Arneson C, Galiè N. Oral treprostinil for the treatment of pulmonary arterial hypertension in patients on background endothelin receptor antagonist and/or phosphodiesterase type 5 inhibitor therapy (the FREEDOM-C study): a randomized controlled trial. Chest. 2012 Dec;142(6):1383-1390. doi: 10.1378/chest.11-2212. PMID: 22628490.	No subgroup analyses reported
Oudiz RJ, Brundage BH, Galiè N, Ghofrani HA, Simonneau G, Botros FT, Chan M, Beardsworth A, Barst RJ; PHIRST Study Group. Tadalafil for the treatment of pulmonary arterial hypertension: a double-blind 52-week uncontrolled extension study. J Am Coll Cardiol. 2012 Aug 21;60(8):768-74. doi: 10.1016/j.jacc.2012.05.004. Epub 2012 Jul 18. PMID: 22818063.	No subgroup analyses reported
Dong MF, Ma ZS, Ma SJ, Chai SD, Tang PZ, Yao DK, Wang L. Effect of prostaglandin E1 on pulmonary arterial hypertension following corrective surgery for congenital heart disease. J Cardiovasc Pharmacol Ther. 2012 Sep;17(3):303-7. doi: 10.1177/1074248411429966. Epub 2011 Dec 27. PMID: 22203134.	No subgroup analyses reported
Attinà TM, Drummond ID, Malatino LS, Maxwell SR, Webb DJ. Phosphodiesterase type 5 inhibition improves arterial stiffness after exercise but not exercise capacity in hypertensive men. Am J Hypertens. 2013 Mar;26(3):342-50. doi: 10.1093/ajh/hps057. Epub 2013 Jan 7. PMID: 23382484.	Patients were not treated for pulmonary hypertension
Denault AY, Pearl RG, Michler RE, Rao V, Tsui SS, Seitelberger R, Cromie M,	No subgroup analyses

Lindberg E, D'Armini AM. Tezosentan and right ventricular failure in patients with pulmonary hypertension undergoing cardiac surgery: the TACTICS trial. J Cardiothorac Vasc Anesth. 2013 Dec;27(6):1212-7. doi: 10.1053/j.jvca.2013.01.023. Epub 2013 Mar 21. PMID: 23523254.	reported
Maron BA, Waxman AB, Opotowsky AR, Gillies H, Blair C, Aghamohammadzadeh R, Loscalzo J, Leopold JA. Effectiveness of spironolactone plus ambrisentan for treatment of pulmonary arterial hypertension (from the [ARIES] study 1 and 2 trials). Am J Cardiol. 2013 Sep 1;112(5):720-5. doi: 10.1016/j.amjcard.2013.04.051. Epub 2013 Jun 7. PMID: 23751938; PMCID: PMC3906683.	Post-hoc analysis of a previously published RCT
Martyniuk TV, Zykov KA, Antonova OIu, Rvacheva AV, Arkhipova OA, Kobal' EA, Masenko VP, Chazova IE. [Effect of bosentan on the clinical status and cellular immunity of patients with idiopathic pulmonary hypertension]. Ter Arkh. 2013;85(1):25-31. Russian. PMID: 23536942.	The article was written in a language other than english/spanish/french
Surie S, Reesink HJ, Marcus JT, van der Plas MN, Kloek JJ, Vonk-Noordegraaf A, Bresser P. Bosentan treatment is associated with improvement of right ventricular function and remodeling in chronic thromboembolic pulmonary hypertension. Clin Cardiol. 2013 Nov;36(11):698-703. doi: 10.1002/clc.22197. Epub 2013 Aug 27. PMID: 24037998; PMCID: PMC6649485.	No subgroup analyses reported
Blanco I, Santos S, Gea J, Güell R, Torres F, Gimeno-Santos E, Rodriguez DA, Vilaró J, Gómez B, Roca J, Barberà JA. Sildenafil to improve respiratory rehabilitation outcomes in COPD: a controlled trial. Eur Respir J. 2013 Oct;42(4):982-92. doi: 10.1183/09031936.00176312. Epub 2013 Feb 21. PMID: 23429918.	No subgroup analyses reported
Elinoff JM, Rame JE, Forfia PR, Hall MK, Sun J, Gharib AM, Abd-Elmoniem K, Graninger G, Harper B, Danner RL, Solomon MA. A pilot study of the effect of spironolactone therapy on exercise capacity and endothelial dysfunction in pulmonary arterial hypertension: study protocol for a randomized controlled trial. Trials. 2013 Apr 2;14:91. doi: 10.1186/1745-6215-14-91. PMID: 23547564; PMCID: PMC3653687.	Clinical Trial protocol
Ersoy O, Boysan E, Unal EU, Yay K, Yener U, Cicekcioglu F, Katircioglu F. Effectiveness of prophylactic levosimendan in high-risk valve surgery patients. Cardiovasc J Afr. 2013 Aug;24(7):260-4. doi: 10.5830/CVJA-2013-047. PMID: 24217302; PMCID: PMC3807672.	Patients were not treated for pulmonary hypertension
Dopp JM, Agapitov AV, Sinkey CA, Haynes WG, Phillips BG. Sildenafil increases sympathetically mediated vascular tone in humans. Am J Hypertens. 2013 Jun;26(6):762-9. doi: 10.1093/ajh/hpt018. Epub 2013 Feb 26. PMID: 23443723; PMCID: PMC3697070.	No subgroup analyses reported
Hoeper MM, Barst RJ, Bourge RC, Feldman J, Frost AE, Galié N, Gómez-Sánchez MA, Grimminger F, Grünig E, Hassoun PM, Morrell NW, Peacock AJ, Satoh T, Simonneau G, Tapson VF, Torres F, Lawrence D, Quinn DA, Ghofrani HA. Imatinib mesylate as add-on therapy for pulmonary arterial	The article did not report on pulmonary hypertension specific-therapy

hypertension: results of the randomized IMPRES study. Circulation. 2013 Mar 12;127(10):1128-38. doi: 10.1161/CIRCULATIONAHA.112.000765. Epub 2013 Feb 12. PMID: 23403476.	
Fukumoto Y, Yamada N, Matsubara H, Mizoguchi M, Uchino K, Yao A, Kihara Y, Kawano M, Watanabe H, Takeda Y, Adachi T, Osanai S, Tanabe N, Inoue T, Kubo A, Ota Y, Fukuda K, Nakano T, Shimokawa H. Double-blind, placebo-controlled clinical trial with a rho-kinase inhibitor in pulmonary arterial hypertension. Circ J. 2013;77(10):2619-25. doi: 10.1253/circj.cj-13-0443. Epub 2013 Aug 3. PMID: 23912836.	No subgroup analyses reported
Raghu G, Behr J, Brown KK, Egan JJ, Kawut SM, Flaherty KR, Martinez FJ, Nathan SD, Wells AU, Collard HR, Costabel U, Richeldi L, de Andrade J, Khalil N, Morrison LD, Lederer DJ, Shao L, Li X, Pedersen PS, Montgomery AB, Chien JW, O'Riordan TG; ARTEMIS-IPF Investigators*. Treatment of idiopathic pulmonary fibrosis with ambrisentan: a parallel, randomized trial. Ann Intern Med. 2013 May 7;158(9):641-9. doi: 10.7326/0003-4819-158-9-201305070-00003. Erratum in: Ann Intern Med. 2014 May 6;160(9):658. PMID: 23648946.	Patients were not treated for pulmonary hypertension
Schuuring MJ, Vis JC, van Dijk AP, van Melle JP, Vliegen HW, Pieper PG, Sieswerda GT, de Bruin-Bon RH, Mulder BJ, Bouma BJ. Impact of bosentan on exercise capacity in adults after the Fontan procedure: a randomized controlled trial. Eur J Heart Fail. 2013 Jun;15(6):690-8. doi: 10.1093/eurjhf/hft017. Epub 2013 Jan 29. PMID: 23361871.	The trial was not design to include pulmonary hypertension patients
Bonderman D, Pretsch I, Steringer-Mascherbauer R, Jansa P, Rosenkranz S, Tufaro C, Bojic A, Lam CSP, Frey R, Ochan Kilama M, Unger S, Roessig L, Lang IM. Acute hemodynamic effects of riociguat in patients with pulmonary hypertension associated with diastolic heart failure (DILATE-1): a randomized, double-blind, placebo-controlled, single-dose study. Chest. 2014 Nov;146(5):1274-1285. doi: 10.1378/chest.14-0106. PMID: 24991733; PMCID: PMC4219342.	No subgroup analyses reported
Dhaun N, Vachiery JL, Benza RL, Naeije R, Hwang LJ, Liu X, Teal S, Webb DJ. Endothelin antagonism and uric acid levels in pulmonary arterial hypertension: clinical associations. J Heart Lung Transplant. 2014 May;33(5):521-7. doi: 10.1016/j.healun.2014.01.853. Epub 2014 Jan 23. PMID: 24656288.	Post-hoc analysis of a previously published RCT
Simonneau G, Rubin LJ, Galiè N, Barst RJ, Fleming TR, Frost A, Engel P, Kramer MR, Serdarevic-Pehar M, Layton GR, Sitbon O, Badesch DB; PACES Study Group. Long-term sildenafil added to intravenous epoprostenol in patients with pulmonary arterial hypertension. J Heart Lung Transplant. 2014 Jul;33(7):689-97. doi: 10.1016/j.healun.2014.02.019. Epub 2014 Feb 22. PMID: 24815795.	No subgroup analyses reported
Sitbon O, Delcroix M, Bergot E, Boonstra AB, Granton J, Langleben D, Subías PE, Galiè N, Pfister T, Lemarié JC, Simonneau G. EPITOME-2: An open-label study assessing the transition to a new formulation of intravenous	No subgroup analyses reported

epoprostenol in patients with pulmonary arterial hypertension. Am Heart J. 2014 Feb;167(2):210-7. doi: 10.1016/j.ahj.2013.08.007. Epub 2013 Oct 3. PMID: 24439982.	
Sun X, Wang K, Wang W, Li B. [Clinical study on sildenafil in treatment of pregnant women with pulmonary arterial hypertension]. Zhonghua Fu Chan Ke Za Zhi. 2014 Jun;49(6):414-8. Chinese. PMID: 25169631.	The article was written in a language other than english/spanish/french
Baughman RP, Culver DA, Cordova FC, Padilla M, Gibson KF, Lower EE, Engel PJ. Bosentan for sarcoidosis-associated pulmonary hypertension: a double-blind placebo controlled randomized trial. Chest. 2014 Apr;145(4):810-817. doi: 10.1378/chest.13-1766. PMID: 24177203.	No subgroup analyses reported
Simonneau G, Galiè N, Jansa P, Meyer GM, Al-Hiti H, Kusic-Pajic A, Lemarié JC, Hoeper MM, Rubin LJ. Long-term results from the EARLY study of bosentan in WHO functional class II pulmonary arterial hypertension patients. Int J Cardiol. 2014 Mar 15;172(2):332-9. doi: 10.1016/j.ijcard.2013.12.179. Epub 2014 Jan 9. PMID: 24525158.	Post-hoc analysis of a previously published RCT
Schumacher DS, Müller-Mottet S, Hasler ED, Hildenbrand FF, Keusch S, Speich R, Bloch KE, Ulrich S. Effect of oxygen and acetazolamide on nocturnal cardiac conduction, repolarization, and arrhythmias in precapillary pulmonary hypertension and sleep-disturbed breathing. Chest. 2014 Nov;146(5):1226-1236. doi: 10.1378/chest.14-0495. PMID: 24991933.	No subgroup analyses reported
Jiang X, Wang YF, Zhao QH, Jiang R, Wu Y, Peng FH, Xu XQ, Wang L, He J, Jing ZC. Acute hemodynamic response of infused fasudil in patients with pulmonary arterial hypertension: a randomized, controlled, crossover study. Int J Cardiol. 2014 Nov 15;177(1):61-5. doi: 10.1016/j.ijcard.2014.09.101. Epub 2014 Sep 28. PMID: 25499341.	No subgroup analyses reported
Feng EZ, Yang SY, Huang NX, Yin H, Zhang Y, Tian ZX. Plasma endothelin-1 and nitric oxide correlate with ligustrazine alleviation of pulmonary artery hypertension in patients of chronic cor pulmonale from high altitude plateau during acute exacerbation. Zhongguo Ying Yong Sheng Li Xue Za Zhi. 2014 Nov;30(6):532-7. PMID: 26016363.	The article was not available
Chin KM, Badesch DB, Robbins IM, Tapson VF, Palevsky HI, Kim NH, Kawut SM, Frost A, Benton WW, Lemarie JC, Bodin F, Rubin LJ, McLaughlin V. Two formulations of epoprostenol sodium in the treatment of pulmonary arterial hypertension: EPITOME-1 (epoprostenol for injection in pulmonary arterial hypertension), a phase IV, open-label, randomized study. Am Heart J. 2014 Feb;167(2):218-225.e1. doi: 10.1016/j.ahj.2013.08.008. Epub 2013 Oct 16. PMID: 24439983.	No subgroup analyses reported
Jiang G, Li B, Zhang G, Xu E, Liu Y, Xu Z. Effects of sildenafil on prognosis in patients with pulmonary hypertension after left-sided valvular surgery. Heart Lung Circ. 2014 Jul;23(7):680-5. doi: 10.1016/j.hlc.2013.12.014. Epub 2014 Jan 31. PMID: 24613188.	No subgroup analyses reported

João BB, do Amaral JL, Bueno RM, Ferez D, Falcão LF, Perez MV, de Oliveira-Júnior IS. Intravenous clonidine administration and its ability to reduce pulmonary arterial pressure in patients undergoing heart surgery. Braz J Anesthesiol. 2014 Jan-Feb;64(1):40-8. doi: 10.1016/j.bjane.2013.03.019. Epub 2013 Dec 17. PMID: 24565387.	No subgroup analyses reported
Goudie AR, Lipworth BJ, Hopkinson PJ, Wei L, Struthers AD. Tadalafil in patients with chronic obstructive pulmonary disease: a randomised, double-blind, parallel-group, placebo-controlled trial. Lancet Respir Med. 2014 Apr;2(4):293-300. doi: 10.1016/S2213-2600(14)70013-X. Epub 2014 Mar 5. PMID: 24717626.	No subgroup analyses reported
Lindegger N, Sidharta PN, Reseski K, Dingemanse J. Macitentan, a dual endothelin receptor antagonist for the treatment of pulmonary arterial hypertension, does not affect cardiac repolarization in healthy subjects. Pulm Pharmacol Ther. 2014 Oct;29(1):41-8. doi: 10.1016/j.pupt.2014.04.007. Epub 2014 May 9. PMID: 24813561.	Patients were not treated for pulmonary hypertension
Bruderer S, Hurst N, Kaufmann P, Dingemanse J. Multiple-dose up-titration study to evaluate the safety, tolerability, pharmacokinetics, and pharmacodynamics of selexipag, an orally available selective prostacyclin receptor agonist, in healthy subjects. Pharmacology. 2014;94(3-4):148-56. doi: 10.1159/000367630. Epub 2014 Sep 25. PMID: 25277144.	Patients were not treated for pulmonary hypertension
Gandhi H, Shah B, Patel R, Toshani R, Pujara J, Kothari J, Shastri N. Effect of preoperative oral sildenafil on severe pulmonary artery hypertension in patients undergoing mitral valve replacement. Indian J Pharmacol. 2014 May-Jun;46(3):281-5. doi: 10.4103/0253-7613.132158. PMID: 24987174; PMCID: PMC4071704.	No subgroup analyses reported
Libri V, Gibbs JS, Pinato DJ, Iddamalgoda T, Khengar RH, Gin-Sing W, Huson L, Anand P. Capsaicin 8% patch for treprostinil subcutaneous infusion site pain in pulmonary hypertension patients. Br J Anaesth. 2014 Feb;112(2):337-47. doi: 10.1093/bja/aet308. Epub 2013 Sep 22. PMID: 24062200.	No subgroup analyses reported
Channick RN, Delcroix M, Ghofrani HA, Hunsche E, Jansa P, Le Brun FO, Mehta S, Pulido T, Rubin LJ, Sastry BKS, Simonneau G, Sitbon O, Souza R, Torbicki A, Galiè N. Effect of macitentan on hospitalizations: results from the SERAPHIN trial. JACC Heart Fail. 2015 Jan;3(1):1-8. doi: 10.1016/j.jchf.2014.07.013. Epub 2014 Nov 11. PMID: 25457902.	Post-hoc analysis of a previously published RCT
D'Armini AM, Ghofrani HA, Kim NH, Mayer E, Morsolini M, Pulido-Zamudio T, Simonneau G, Wilkins MR, Curram J, Davie N, Hoeper MM. Use of responder threshold criteria to evaluate the response to treatment in the phase III CHEST-1 study. J Heart Lung Transplant. 2015 Mar;34(3):348-55. doi: 10.1016/j.healun.2015.02.003. Epub 2015 Feb 16. PMID: 25813765.	Post-hoc analysis of a previously published RCT
Langleben D, Galiè N, He J, Huang Y, Humbert M, Keogh A, Rubin LJ, Zhou D, Curram J, Davie N, Ghofrani HA. Use of clinically relevant responder threshold criteria to evaluate the response to treatment in the phase III	Post-hoc analysis of a previously published RCT

PATENT-1 study. J Heart Lung Transplant. 2015 Mar;34(3):338-47. doi: 10.1016/j.healun.2014.12.001. Epub 2014 Dec 12. PMID: 25703961.	
Simonneau G, Channick RN, Delcroix M, Galiè N, Ghofrani HA, Jansa P, Le Brun FO, Mehta S, Perchenet L, Pulido T, Sastry BK, Sitbon O, Souza R, Torbicki A, Rubin LJ. Incident and prevalent cohorts with pulmonary arterial hypertension: insight from SERAPHIN. Eur Respir J. 2015 Dec;46(6):1711-20. doi: 10.1183/13993003.00364-2015. Epub 2015 Oct 22. PMID: 26493786; PMCID: PMC4664609.	Post-hoc analysis of a previously published RCT
Raghu G, Nathan SD, Behr J, Brown KK, Egan JJ, Kawut SM, Flaherty KR, Martinez FJ, Wells AU, Shao L, Zhou H, Henig N, Szwarcberg J, Gillies H, Montgomery AB, O'Riordan TG. Pulmonary hypertension in idiopathic pulmonary fibrosis with mild-to-moderate restriction. Eur Respir J. 2015 Nov;46(5):1370-7. doi: 10.1183/13993003.01537-2014. Epub 2015 Aug 6. PMID: 26250495.	No subgroup analyses reported
Rosenkranz S, Ghofrani HA, Beghetti M, Ivy D, Frey R, Fritsch A, Weimann G, Saleh S, Apitz C. Riociguat for pulmonary arterial hypertension associated with congenital heart disease. Heart. 2015 Nov;101(22):1792-9. doi: 10.1136/heartjnl-2015-307832. Epub 2015 Jul 1. PMID: 26135803; PMCID: PMC4680166.	Post-hoc analysis of a previously published RCT
Vlachogeorgos GS, Daskalopoulos N, Blatsiotis P, Kourbeti IS, Mantas I, Stathopoulos GT. Bosentan for patients with echocardiographic evidence of pulmonary hypertension due to long-standing rheumatic mitral stenosis. Hellenic J Cardiol. 2015 Jan-Feb;56(1):36-43. PMID: 25701970.	The article was not available
Frost AE, Barst RJ, Hoeper MM, Chang HJ, Frantz RP, Fukumoto Y, Galié N, Hassoun PM, Klose H, Matsubara H, Morrell NW, Peacock AJ, Pfeifer M, Simonneau G, Tapson VF, Torres F, Dario Vizza C, Lawrence D, Yang W, Felser JM, Quinn DA, Ghofrani HA. Long-term safety and efficacy of imatinib in pulmonary arterial hypertension. J Heart Lung Transplant. 2015 Nov;34(11):1366-75. doi: 10.1016/j.healun.2015.05.025. Epub 2015 Jun 11. PMID: 26210752.	No subgroup analyses reported
Galiè N, Müller K, Scalise AV, Grünig E. PATENT PLUS: a blinded, randomised and extension study of riociguat plus sildenafil in pulmonary arterial hypertension. Eur Respir J. 2015 May;45(5):1314-22. doi: 10.1183/09031936.00105914. Epub 2015 Feb 5. PMID: 25657022.	No subgroup analyses reported
Simonneau G, D'Armini AM, Ghofrani HA, Grimminger F, Hoeper MM, Jansa P, Kim NH, Wang C, Wilkins MR, Fritsch A, Davie N, Colorado P, Mayer E. Riociguat for the treatment of chronic thromboembolic pulmonary hypertension: a long-term extension study (CHEST-2). Eur Respir J. 2015 May;45(5):1293-302. doi: 10.1183/09031936.00087114. Epub 2014 Nov 13. PMID: 25395036.	No subgroup analyses reported
Rubin LJ, Galiè N, Grimminger F, Grünig E, Humbert M, Jing ZC, Keogh A, Langleben D, Fritsch A, Menezes F, Davie N, Ghofrani HA. Riociguat for the treatment of pulmonary arterial hypertension: a long-term extension study	No subgroup analyses reported

(PATENT-2). Eur Respir J. 2015 May;45(5):1303-13. doi: 10.1183/09031936.00090614. Epub 2015 Jan 22. PMID: 25614164.	
Querejeta Roca G, Campbell P, Claggett B, Vazir A, Quinn D, Solomon SD, Shah AM. Impact of lowering pulmonary vascular resistance on right and left ventricular deformation in pulmonary arterial hypertension. Eur J Heart Fail. 2015 Jan;17(1):63-73. doi: 10.1002/ejhf.177. Epub 2014 Nov 4. PMID: 25367310; PMCID: PMC5551563.	Post-hoc analysis of a previously published RCT
Shah AM, Campbell P, Rocha GQ, Peacock A, Barst RJ, Quinn D, Solomon SD; IMPRES Investigators. Effect of imatinib as add-on therapy on echocardiographic measures of right ventricular function in patients with significant pulmonary arterial hypertension. Eur Heart J. 2015 Mar 7;36(10):623-32. doi: 10.1093/eurheartj/ehu035. Epub 2014 Feb 23. PMID: 24566799; PMCID: PMC4481574.	No subgroup analyses reported
Ulrich S, Keusch S, Hildenbrand FF, Lo Cascio C, Huber LC, Tanner FC, Speich R, Bloch KE. Effect of nocturnal oxygen and acetazolamide on exercise performance in patients with pre-capillary pulmonary hypertension and sleep-disturbed breathing: randomized, double-blind, cross-over trial. Eur Heart J. 2015 Mar 7;36(10):615-23. doi: 10.1093/eurheartj/eht540. Epub 2013 Dec 23. PMID: 24366914.	The article did not report on pulmonary hypertension specific-therapy
Mishra A, Kumar B, Dutta V, Arya VK, Mishra AK. Comparative Effect of Levosimendan and Milrinone in Cardiac Surgery Patients With Pulmonary Hypertension and Left Ventricular Dysfunction. J Cardiothorac Vasc Anesth. 2016 Jun;30(3):639-46. doi: 10.1053/j.jvca.2016.01.015. Epub 2016 Jan 12. PMID: 27321790.	No subgroup analyses reported
Abe S, Ishida K, Masuda M, Ueda H, Kohno H, Matsuura K, Tamura Y, Watanabe M, Matsumiya G. A prospective, randomized study of inhaled prostacyclin versus nitric oxide in patients with residual pulmonary hypertension after pulmonary endarterectomy. Gen Thorac Cardiovasc Surg. 2017 Mar;65(3):153-159. doi: 10.1007/s11748-016-0724-2. Epub 2016 Oct 25. PMID: 27783213; PMCID: PMC5331109.	No subgroup analyses reported
Coghlan JG, Galiè N, Barberà JA, Frost AE, Ghofrani HA, Hoeper MM, Kuwana M, McLaughlin VV, Peacock AJ, Simonneau G, Vachiéry JL, Blair C, Gillies H, Miller KL, Harris JHN, Langley J, Rubin LJ; AMBITION investigators. Initial combination therapy with ambrisentan and tadalafil in connective tissue disease-associated pulmonary arterial hypertension (CTD-PAH): subgroup analysis from the AMBITION trial. Ann Rheum Dis. 2017 Jul;76(7):1219-1227. doi: 10.1136/annrheumdis-2016-210236. Epub 2016 Dec 30. PMID: 28039187; PMCID: PMC5530350.	Post-hoc analysis of a previously published RCT
Galiè N, Denton CP, Dardi F, Manes A, Mazzanti G, Li B, Varanese L, Esler A, Harmon C, Palazzini M. Tadalafil in idiopathic or heritable pulmonary arterial hypertension (PAH) compared to PAH associated with connective tissue disease. Int J Cardiol. 2017 May 15;235:67-72. doi: 10.1016/j.ijcard.2017.02.094. Epub 2017 Feb 22. PMID: 28279499.	Post-hoc analysis of a previously published RCT

Han X, Zhang Y, Dong L, Fang L, Chai Y, Niu M, Yu Y, Liu L, Yang X, Qu S, Li S. Treatment of Pulmonary Arterial Hypertension Using Initial Combination Therapy of Bosentan and Iloprost. Respir Care. 2017 Apr;62(4):489-496. doi: 10.4187/respcare.05280. Epub 2017 Jan 24. PMID: 28119496.	No subgroup analyses reported
Humbert M, Coghlan JG, Ghofrani HA, Grimminger F, He JG, Riemekasten G, Vizza CD, Boeckenhoff A, Meier C, de Oliveira Pena J, Denton CP. Riociguat for the treatment of pulmonary arterial hypertension associated with connective tissue disease: results from PATENT-1 and PATENT-2. Ann Rheum Dis. 2017 Feb;76(2):422-426. doi: 10.1136/annrheumdis-2015-209087. Epub 2016 Jul 25. PMID: 27457511; PMCID: PMC5284330.	Post-hoc analysis of a previously published RCT
Koller B, Steringer-Mascherbauer R, Ebner CH, Weber T, Ammer M, Eichinger J, Pretsch I, Herold M, Schwaiger J, Ulmer H, Grander W. Pilot Study of Endothelin Receptor Blockade in Heart Failure with Diastolic Dysfunction and Pulmonary Hypertension (BADDHY-Trial). Heart Lung Circ. 2017 May;26(5):433-441. doi: 10.1016/j.hlc.2016.09.004. Epub 2016 Sep 28. PMID: 27816421.	No subgroup analyses reported
Shrestha SK , Srivastava B , Karki M , Khatri DB , Pradhan RM . Effect of Sildenafil Citrate on Pulmonary Arterial Systolic Pressure and Sub-maximal Exercise Capacity in Chronic Obstructive Pulmonary Disease. Kathmandu Univ Med J (KUMJ). 2017 Oct.-Dec.;15(60):271-278. PMID: 30580340.	The article was not available
Tanaka Y, Hino M, Gemma A. Potential benefit of bosentan therapy in borderline or less severe pulmonary hypertension secondary to idiopathic pulmonary fibrosis-an interim analysis of results from a prospective, single-center, randomized, parallel-group study. BMC Pulm Med. 2017 Dec 13;17(1):200. doi: 10.1186/s12890-017-0523-2. PMID: 29237441; PMCID: PMC5729252.	Subgroup analyses missing data
Vitulo P, Stanziola A, Confalonieri M, Libertucci D, Oggionni T, Rottoli P, Paciocco G, Tuzzolino F, Martino L, Beretta M, Callari A, Amaducci A, Badagliacca R, Poscia R, Meloni F, Refini RM, Geri P, Baldi S, Ghio S, D'Alto M, Argiento P, Sofia M, Guardamagna M, Pezzuto B, Vizza CD. Sildenafil in severe pulmonary hypertension associated with chronic obstructive pulmonary disease: A randomized controlled multicenter clinical trial. J Heart Lung Transplant. 2017 Feb;36(2):166-174. doi: 10.1016/j.healun.2016.04.010. Epub 2016 May 7. PMID: 27329400.	No subgroup analyses reported
Jalalian R, Moghadamnia AA, Tamaddoni A, Khafri S, Iranian M. Comparing the Efficacy of Tadalafil Versus Placebo on Pulmonary Artery Systolic Pressure and Right Ventricular Function in Patients with Beta-Thalassaemia Intermedia. Heart Lung Circ. 2017 Jul;26(7):677-683. doi: 10.1016/j.hlc.2016.11.003. Epub 2016 Dec 2. PMID: 27989690.	No subgroup analyses reported
Gaine S, Chin K, Coghlan G, Channick R, Di Scala L, Galiè N, Ghofrani HA, Lang IM, McLaughlin V, Preiss R, Rubin LJ, Simonneau G, Sitbon O, Tapson VF, Hoeper MM. Selexipag for the treatment of connective tissue disease-associated pulmonary arterial hypertension. Eur Respir J. 2017 Aug	Post-hoc analysis of a previously published RCT

17;50(2):1602493. doi: 10.1183/13993003.02493-2016. PMID: 28818881; PMCID: PMC5593379.	
Galiè N, Grimminger F, Grünig E, Hoeper MM, Humbert M, Jing ZC, Keogh AM, Langleben D, Rubin LJ, Fritsch A, Davie N, Ghofrani HA. Comparison of hemodynamic parameters in treatment-naïve and pre-treated patients with pulmonary arterial hypertension in the randomized phase III PATENT-1 study. J Heart Lung Transplant. 2017 May;36(5):509-519. doi: 10.1016/j.healun.2016.12.012. Epub 2016 Dec 24. PMID: 28190787.	Post-hoc analysis of a previously published RCT
Liu LC, Hummel YM, van der Meer P, Berger RM, Damman K, van Veldhuisen DJ, Voors AA, Hoendermis ES. Effects of sildenafil on cardiac structure and function, cardiopulmonary exercise testing and health-related quality of life measures in heart failure patients with preserved ejection fraction and pulmonary hypertension. Eur J Heart Fail. 2017 Jan;19(1):116-125. doi: 10.1002/ejhf.662. Epub 2016 Nov 21. PMID: 27873388.	No subgroup analyses reported
Distler O, Pope J, Denton C, Allanore Y, Matucci-Cerinic M, de Oliveira Pena J, Khanna D. RISE-SSc: Riociguat in diffuse cutaneous systemic sclerosis. Respir Med. 2017 Jan;122 Suppl 1:S14-S17. doi: 10.1016/j.rmed.2016.09.011. Epub 2016 Sep 28. PMID: 27746061.	Clinical Trial protocol
Herbert S, Gin-Sing W, Howard L, Tulloh RMR. Early Experience of Macitentan for Pulmonary Arterial Hypertension in Adult Congenital Heart Disease. Heart Lung Circ. 2017 Oct;26(10):1113-1116. doi: 10.1016/j.hlc.2016.12.011. Epub 2017 Feb 6. PMID: 28237536.	No subgroup analyses reported
Kawut SM, Archer-Chicko CL, DeMichele A, Fritz JS, Klinger JR, Ky B, Palevsky HI, Palmisciano AJ, Patel M, Pinder D, Propert KJ, Smith KA, Stanczyk F, Tracy R, Vaidya A, Whittenhall ME, Ventetuolo CE. Anastrozole in Pulmonary Arterial Hypertension. A Randomized, Double-Blind, Placebo-controlled Trial. Am J Respir Crit Care Med. 2017 Feb 1;195(3):360-368. doi: 10.1164/rccm.201605-1024OC. PMID: 27602993; PMCID: PMC5328182.	The article did not report on pulmonary hypertension specific-therapy
Vachiéry JL, Delcroix M, Al-Hiti H, Efficace M, Hutyra M, Lack G, Papadakis K, Rubin LJ. Macitentan in pulmonary hypertension due to left ventricular dysfunction. Eur Respir J. 2018 Feb 7;51(2):1701886. doi: 10.1183/13993003.01886-2017. PMID: 29437943.	No subgroup analyses reported
Marra AM, Halank M, Benjamin N, Bossone E, Cittadini A, Eichstaedt CA, Egenlauf B, Harutyunova S, Fischer C, Gall H, Ghofrani HA, Hoeper MM, Lange TJ, Olsson KM, Klose H, Grünig E. Right ventricular size and function under riociguat in pulmonary arterial hypertension and chronic thromboembolic pulmonary hypertension (the RIVER study). Respir Res. 2018 Dec 19;19(1):258. doi: 10.1186/s12931-018-0957-y. PMID: 30567595; PMCID: PMC6299931.	No subgroup analyses reported
Behr J, Nathan SD, Harari S, Wuyts W, Kirchgaessler KU, Bengus M, Gilberg F, Wells AU. Sildenafil added to pirfenidone in patients with advanced idiopathic pulmonary fibrosis and risk of pulmonary hypertension: A Phase IIb, randomised, double-blind, placebo-controlled study - Rationale and	Clinical Trial protocol

study design. Respir Med. 2018 May;138:13-20. doi: 10.1016/j.rmed.2018.03.019. Epub 2018 Mar 16. PMID: 29724385.	
Ruan HY, Zhang YG, Liu R. [Acute effects of intravenous fasudil with different dosage on patients with congenital heart defects and severe pulmonary arterial hypertension]. Zhonghua Yi Xue Za Zhi. 2018 Mar 6;98(9):678-681. Chinese. doi: 10.3760/cma.j.issn.0376-2491.2018.09.011. PMID: 29534403.	No subgroup analyses reported
Eskandr AM, Metwally AA, Abu Elkassem MS, Sadik SA, Elmiligy AE, Mourad M, Hussein L. Dobutamine and Nitroglycerin Versus Milrinone for Perioperative Management of Pulmonary Hypertension in Mitral Valve Surgery. A Randomized Controlled Study. J Cardiothorac Vasc Anesth. 2018 Dec;32(6):2540-2546. doi: 10.1053/j.jvca.2018.04.032. Epub 2018 Apr 18. PMID: 29880427.	No subgroup analyses reported
Kundra TS, Nagaraja PS, Bharathi KS, Kaur P, Manjunatha N. Inhaled levosimendan versus intravenous levosimendan in patients with pulmonary hypertension undergoing mitral valve replacement. Ann Card Anaesth. 2018 Jul-Sep;21(3):328-332. doi: 10.4103/aca.ACA_19_18. PMID: 30052230; PMCID: PMC6078018.	No subgroup analyses reported
Boyko AN, Bosenko LP, Vasilovskiy VV, Volkova LI, Zakharova MN, Kotov SV, Lekomtseva EV, Negrich TI, Parshina EV, Patrusheva OP, Prokopenko SV, Sazonov DV, Timchenko LV, Trinitatskiy YV, Khabirov FA, Khavunka MY, Chichanovskaya LV, Sherman MA, Ivanov RA, Lin'kova YN, Stukalina EY, Zinkina-Orikhan AV, Obukhova IG. Éffektivnost', perenosimost' i bezopasnost' terapii preparatom teberif: rezul'taty dvukhletnego klinicheskogo issledovaniia u patsientov s remittiruiushchim rasseiannym sklerozom, ranee ne poluchavshikh PITRS, i pri perekliuchenii s drugogo interferona β-1a [Efficacy, tolerability and safety of the treatment with teberif: the results of a 2-year randomized clinical trial of treatment naïve patients with remitting multiple sclerosis, who have not received DMT, after switching from other interferon β-1a]. Zh Nevrol Psikhiatr Im S S Korsakova. 2019;119(2. Vyp. 2):73-85. Russian. doi: 10.17116/jnevro20191192273. PMID: 31156245.	The article was written in a language other than english/spanish/french
Clavé MM, Maeda NY, Thomaz AM, Bydlowski SP, Lopes AA. Phosphodiesterase type 5 inhibitors improve microvascular dysfunction markers in pulmonary arterial hypertension associated with congenital heart disease. Congenit Heart Dis. 2019 Mar;14(2):246-255. doi: 10.1111/chd.12688. Epub 2018 Oct 20. PMID: 30343508.	No subgroup analyses reported
Frost A, Janmohamed M, Fritz JS, McConnell JW, Poch D, Fortin TA, Miller CE, Chin KM, Fisher M, Eggert M, McEvoy C, Benza RL, Farber HW, Kim NH, Pfister T, Shiraga Y, McLaughlin V. Safety and tolerability of transition from inhaled treprostinil to oral selexipag in pulmonary arterial hypertension: Results from the TRANSIT-1 study. J Heart Lung Transplant. 2019 Jan;38(1):43-50. doi: 10.1016/j.healun.2018.09.003. Epub 2018 Sep 12. PMID: 30391194.	No subgroup analyses reported

Hobbs AJ, Moyes AJ, Baliga RS, Ghedia D, Ochiel R, Sylvestre Y, Doré CJ, Chowdhury K, Maclagan K, Quartly HL, Sofat R, Smit A, Schreiber BE, Coghlan GJ, MacAllister RJ. Neprilysin inhibition for pulmonary arterial hypertension: a randomized, double-blind, placebo-controlled, proof-of-concept trial. Br J Pharmacol. 2019 May;176(9):1251-1267. doi: 10.1111/bph.14621. Epub 2019 Mar 31. PMID: 30761523; PMCID: PMC7651846.	No subgroup analyses reported
McLaughlin VV, Vachiery JL, Oudiz RJ, Rosenkranz S, Galiè N, Barberà JA, Frost AE, Ghofrani HA, Peacock AJ, Simonneau G, Rubin LJ, Blair C, Langley J, Hoeper MM; AMBITION Study Group. Patients with pulmonary arterial hypertension with and without cardiovascular risk factors: Results from the AMBITION trial. J Heart Lung Transplant. 2019 Dec;38(12):1286-1295. doi: 10.1016/j.healun.2019.09.010. Epub 2019 Sep 17. PMID: 31648845.	No subgroup analyses reported
Ruan H, Zhang Y, Liu R, Yang X. The acute effects of 30 mg vs 60 mg of intravenous Fasudil on patients with congenital heart defects and severe pulmonary arterial hypertension. Congenit Heart Dis. 2019 Jul;14(4):645-650. doi: 10.1111/chd.12764. Epub 2019 Jun 5. PMID: 31166081.	No subgroup analyses reported
Torres F, Farber H, Ristic A, McLaughlin V, Adams J, Zhang J, Klassen P, Shanahan W, Grundy J, Hoffmann I, Cabell C, Escribano Subías P, Sood N, Keogh A, D'Souza G, Rubin L. Efficacy and safety of ralinepag, a novel oral IP agonist, in PAH patients on mono or dual background therapy: results from a phase 2 randomised, parallel group, placebo-controlled trial. Eur Respir J. 2019 Oct 10;54(4):1901030. doi: 10.1183/13993003.01030-2019. PMID: 31391223.	No subgroup analyses reported
White RJ, Vonk-Noordegraaf A, Rosenkranz S, Oudiz RJ, McLaughlin VV, Hoeper MM, Grünig E, Ghofrani HA, Chakinala MM, Barberà JA, Blair C, Langley J, Frost AE. Clinical outcomes stratified by baseline functional class after initial combination therapy for pulmonary arterial hypertension. Respir Res. 2019 Sep 12;20(1):208. doi: 10.1186/s12931-019-1180-1. PMID: 31511080; PMCID: PMC6739949.	Post-hoc analysis of a previously published RCT
Winterhalter M, Rex S, Stoppe C, Kienbaum P, Müller HH, Kaufmann I, Kuppe H, Dongas A, Zwissler B; ILOCARD Investigators. Effect of iloprost inhalation on postoperative outcome in high-risk cardiac surgical patients: a prospective randomized-controlled multicentre trial (ILOCARD). Can J Anaesth. 2019 Aug;66(8):907-920. English. doi: 10.1007/s12630-019-01309-8. Epub 2019 Feb 12. PMID: 30756339.	Patients were not treated for pulmonary hypertension
Ram E, Sternik L, Klempfner R, Eldar M, Goldenberg I, Peled Y, Raanani E, Kogan A. Sildenafil for Pulmonary Hypertension in the Early Postoperative Period After Mitral Valve Surgery. J Cardiothorac Vasc Anesth. 2019 Jun;33(6):1648-1656. doi: 10.1053/j.jvca.2018.12.023. Epub 2018 Dec 25. PMID: 30685151.	No subgroup analyses reported
Sadushi-Kolici R, Jansa P, Kopec G, Torbicki A, Skoro-Sajer N, Campean IA, Halank M, Simkova I, Karlocai K, Steringer-Mascherbauer R, Samarzija M,	No subgroup analyses reported

Salobir B, Klepetko W, Lindner J, Lang IM. Subcutaneous treprostinil for the treatment of severe non-operable chronic thromboembolic pulmonary hypertension (CTREPH): a double-blind, phase 3, randomised controlled trial. Lancet Respir Med. 2019 Mar;7(3):239-248. doi: 10.1016/S2213-2600(18)30367-9. Epub 2018 Nov 23. PMID: 30477763.	
Nathan SD, Behr J, Collard HR, Cottin V, Hoeper MM, Martinez FJ, Corte TJ, Keogh AM, Leuchte H, Mogulkoc N, Ulrich S, Wuyts WA, Yao Z, Boateng F, Wells AU. Riociguat for idiopathic interstitial pneumonia-associated pulmonary hypertension (RISE-IIP): a randomised, placebo-controlled phase 2b study. Lancet Respir Med. 2019 Sep;7(9):780-790. doi: 10.1016/S2213-2600(19)30250-4. Epub 2019 Aug 12. PMID: 31416769.	Subgroup analyses missing data
Pan Z, Marra AM, Benjamin N, Eichstaedt CA, Blank N, Bossone E, Cittadini A, Coghlan G, Denton CP, Distler O, Egenlauf B, Fischer C, Harutyunova S, Xanthouli P, Lorenz HM, Grünig E. Early treatment with ambrisentan of mildly elevated mean pulmonary arterial pressure associated with systemic sclerosis: a randomized, controlled, double-blind, parallel group study (EDITA study). Arthritis Res Ther. 2019 Oct 26;21(1):217. doi: 10.1186/s13075-019-1981-0. PMID: 31655622; PMCID: PMC6815440.	No subgroup analyses reported
Huang CY, Lee JK, Chen ZW, Cheng JF, Chen SY, Lin LY, Wu CK. Inhaled Prostacyclin on Exercise Echocardiographic Cardiac Function in Preserved Ejection Fraction Heart Failure. Med Sci Sports Exerc. 2020 Feb;52(2):269-277. doi: 10.1249/MSS.0000000000002145. PMID: 31479003.	No subgroup analyses reported
Nashat H, Kempny A, Harries C, Dormand N, Alonso-Gonzalez R, Price LC, Gatzoulis MA, Dimopoulos K, Wort SJ. A single-centre, placebo-controlled, double-blind randomised cross-over study of nebulised iloprost in patients with Eisenmenger syndrome: A pilot study. Int J Cardiol. 2020 Jan 15;299:131-135. doi: 10.1016/j.ijcard.2019.07.004. Epub 2019 Jul 4. PMID: 31371115.	No subgroup analyses reported
Khanna D, Allanore Y, Denton CP, Kuwana M, Matucci-Cerinic M, Pope JE, Atsumi T, Bečvář R, Czirják L, Hachulla E, Ishii T, Ishikawa O, Johnson SR, De Langhe E, Stagnaro C, Riccieri V, Schiopu E, Silver RM, Smith V, Steen V, Stevens W, Szücs G, Truchetet ME, Wosnitza M, Laapas K, de Oliveira Pena J, Yao Z, Kramer F, Distler O. Riociguat in patients with early diffuse cutaneous systemic sclerosis (RISE-SSc): randomised, double-blind, placebo-controlled multicentre trial. Ann Rheum Dis. 2020 May;79(5):618-625. doi: 10.1136/annrheumdis-2019-216823. PMID: 32299845; PMCID: PMC7213318.	No subgroup analyses reported
