# Supplementary material for: Subgroup Analysis in Pulmonary Hypertension-Specific Therapy Clinical Trials: A Systematic Review
Source: J Pers Med. 2022 May 25;12(6):863. doi: 10.3390/jpm12060863 (PMC9224970; doi:10.3390/jpm12060863)
Supplement: Supplementary file 1 [file jpm-12-00863-s001.zip › Supplemental File S5.pdf]

Supplemental File S5. Risk of Bias graphs within studies and across studies.

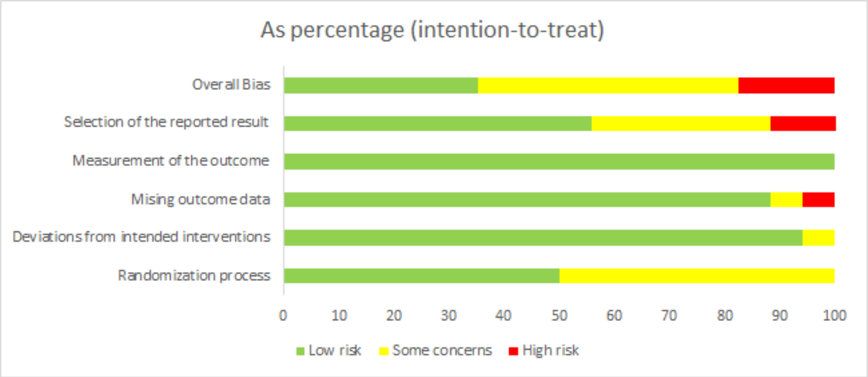

Unique ID	D1	D2	D3	D4	D5	Overall
Galie 2002	!	+	+	+	+	!
Ghofrani 2002	+	+	+	+	-	-
Rubin 2002	!	+	+	+	+	!
Olschewski 2002	!	!	-	+	!	-
Galie 2005 ?	!	+	+	+	!	!
Galie 2005	!	+	+	+	+	!
Jais 2008	!	+	+	+	!	!
Galie 2008	+	+	+	+	+	+
Simonneau 2008	+	+	+	+	+	+
Benza 2008	!	+	+	+	-	-
Galie 2009	!	+	+	+	+	+
Barst 2011	+	+	+	+	-	-
Sandoval 2012	!	+	!	+	!	!
Tapson 2013	!	+	+	+	+	!
Bonderman 2013	!	+	+	+	-	-
Jing 2013	!	+	+	+	+	!
Pulido 2013	+	+	+	+	+	+
Ghofrani 2013	+	+	+	+	+	+
Ghofrani 2013a	+	+	+	+	+	+
Corte 2014	+	+	+	+	+	+
Zhuang 2014	!	!	+	+	+	!
Sitbon 2015	+	+	+	+	+	+
Galie 2015	+	+	+	+	+	+
Hoendermis 2015	+	+	+	+	+	+
McLaughlin 2015	!	+	-	+	+	-
Vizza 2017	!	+	+	+	!	!
Ghofrani 2017	+	+	+	+	+	+
Kim 2017	+	+	+	+	!	!
Metha 2017	+	+	+	+	!	!
Coghlan 2018	+	+	+	+	!	!
Bermejo 2018	!	+	+	+	!	!

D1 Randomisation process

D2 Deviations from the intended interventions

D3 Missing outcome data

D4 Measurement of the outcome

D5 Selection of the reported result

+ Low risk

! Some concerns

- High risk
